# A low-cost open-source SNP genotyping platform for association mapping applications

**DOI:** 10.1186/gb-2005-6-12-r105

**Published:** 2005-12-02

**Authors:** Stuart J Macdonald, Tomi Pastinen, Anne Genissel, Theodore W Cornforth, Anthony D Long

**Affiliations:** 1 Department of Ecology and Evolutionary Biology, University of California Irvine, CA 92697-2525, USA; 2 McGill University and Genome Québec Innovation Centre, 740 Docteur Penfield Avenue, Montreal, Québec H3A 1A4, Canada; 3 Section of Evolution and Ecology, University of California Davis, Davis, CA 95616, USA; 4 Institute of Neuroscience, 1254 University of Oregon, Eugene, Oregon 97403-1254, USA

## Abstract

Association mapping aimed at identifying DNA polymorphisms that contribute to variation in complex traits entails genotyping a large number of single-nucleotide polymorphisms (SNPs) in a very large panel of individuals. Few technologies, however, provide inexpensive high-throughput genotyping. Here, we present an efficient approach developed specifically for genotyping large fixed panels of diploid individuals. The cost-effective, open-source nature of our methodology may make it particularly attractive to those working in nonmodel systems.

## Abstract 2

An efficient, cost-effective and open-source approach is described for high-throughput genotyping of large fixed panels of diploid individuals.

## Background

Understanding the genetic architecture of complex polygenic traits is a fundamental goal of modern biological and medical research, and the currently favored experimental paradigm is association mapping (reviewed by Carlson *et al*. [1]). Association studies genotype a dense set of single nucleotide polymorphisms (SNPs) in a large panel of individuals and test each SNP, or set of local haplotypes constructed from the SNP data, for a phenotype/disease association. A significant association at a query SNP suggests it is the causal polymorphism, or is in strong linkage disequilibrium with the causal site [2-4]. As a class, SNPs represent the most abundant form of genetic variation, with approximately two intermediate frequency SNPs per kilobase in the human genome [5]. Thus, even with some *a priori *knowledge of a candidate gene region contributing to a disease phenotype, a large number of SNPs need to be genotyped in an association mapping study to ensure one of the genotyped SNPs is causative or is in strong linkage disequilibrium with the causative site. It is also important that SNPs are genotyped in a very large panel of individuals to provide sufficient power to detect variants that may have only subtle phenotypic effects. Studies suggest panel sizes of much larger than 1,000 individuals are required to achieve modest power to detect associations if they are present [4,6,7].

A plethora of SNP genotyping platforms is currently available (reviewed by Kwok [8] and Syvänen [9,10]). Several excellent technologies genotype thousands of sites simultaneously, for example, Perlegen Sciences Inc. genotyping arrays [11], Affymetrix Inc. GeneChip arrays [12-15], and Illumina Inc. BeadArray technology coupled with the GoldenGate genotyping assay [16-18]. Such methods may not be cost effective for genotyping a large panel for a more modest number of SNPs. Other methods, such as Biotage Inc. Pyrosequencing [19,20], Applied Biosystems TaqMan approach [21,22], and certain template-directed single base extension methods [23], are readily applied to a large panel, but optimal probes must be designed for each SNP, and multiplexing may be difficult or impossible. Between these two extremes (ultra-high multiplexing and low/no multiplexing) it is difficult to identify the right genotyping system to efficiently and cost-effectively generate genotypes for a very large sample (thousands of individuals) for an intermediate number of SNPs (tens to hundreds of sites). This may be particularly true for those working on nonhuman systems. For human biologists there are several 'off-the-shelf' commercial genotyping solutions. For instance, Affymetrix produce GeneChip 100K arrays [15], offering a fixed set of 100,000 SNPs distributed across the human genome, and pre-designed Applied Biosystems TaqMan assays [21,22] are available for over two million human SNPs. Outside of humans, however, readily available inexpensive genotyping solutions are unavailable, and are likely to remain so for some time. Thus, even as the cost of sequencing continues to fall, and the number of SNPs identified in a variety of nonhuman organisms increases, researchers in nonmodel systems may have difficulty identifying a genotyping system that suits their needs.

Here we describe a low cost SNP genotyping platform developed specifically for large panel sizes and an intermediate number of SNPs. Our platform allows hundreds of SNPs and insertion/deletion polymorphisms to be genotyped in thousands of individuals, and thus may be particularly appropriate for dissecting complex traits in cases where the search space is limited to a set of candidate gene regions. In common with many SNP genotyping systems used today, our method is an amalgam of well-known, robust techniques, including PCR [24,25], hybridization [26], and the oligonucleotide ligation assay (OLA) [27]. We employ a multiplexed OLA, ligation-dependent amplification of allele-specific products, and array-based allele-detection. Our approach builds on the work of Gerry *et al*. [28], and shares a number of similarities with commercial technologies, including Keygene's SNPWave [29], and Applied Biosystem's SNPlex [22], yet offers potentially higher throughput as it detects allele-specific products via arrays as opposed to size separation using a capillary sequencing instrument. Our method is cost-effective for very large panels of individuals (less than $0.03/genotype), does not entail purchasing expensive proprietary equipment or modified long oligonucleotides, and allows robust, parallelized genotyping of many SNPs with limited sample handling. In pursuit of an open-source genotyping system, in the manner of the Brown-style [30] microarray technology, we have made all details of the method available in the Additional data files. These include plans for constructing a Cartesian arraying robot, the associated controller software, detailed protocols for the molecular biology steps, and software for designing the SNP assays and for calling genotypes.

## Results and discussion

We designed SNP genotyping assays for 156 biallelic polymorphisms in the *Enhancer of split *locus and 12 SNPs upstream of the *hairy *locus in *Drosophila melanogaster*. These 168 polymorphisms were genotyped in a fixed panel of approximately 2,000 flies from a single outbred population. DNA extracted from the fly population was arrayed into six 384-well plates, and used as template for 12 long (2 to 3 kb) PCR amplicons, which in turn were used as template for multiplexed OLA reactions. Twenty 8-plex OLA reactions were performed on single 2 to 3 kb amplicons as template, and one 8-plex reaction used two pooled PCR amplicons as template. Following amplification of the products of ligation, each sample was printed in duplicate onto nylon membranes. This resulted in a set of 10 membranes holding SNPs incorporating barcode pairs 01 to 08, and a set of 11 membranes holding SNPs incorporating barcode pairs 09 to 16. Within each set, membranes were combined and sequentially hybridized with the appropriate 16 labeled barcodes to generate the genotype data. The background-subtracted array intensity data are provided in Additional data files 9 (replicate spot 1) and 10 (replicate spot 2), and the genotypes assigned to the individuals are given in Additional data file 11.

### Sensitivity to secondary SNPs

All OLA-based genotyping approaches rely on oligos binding to a small region flanking the query SNP. If this flanking region harbors a minor allele at a SNP other than the query SNP, binding and subsequent ligation efficiency could be hindered if designed OLA oligonucleotides only match the major allele at this secondary SNP. Thus, a secondary SNP could cause the entire genotyping assay to fail, or in double heterozygotes for the query and secondary SNPs, result in incorrect genotype assignment. Because full resequencing data were available around each of the query SNPs (16 alleles for *Enhancer of split *[31] and 10 alleles for *hairy *[32]), we were able to assess the sensitivity of OLA-based genotyping to secondary SNPs in oligo binding regions.

When the resequencing data indicate that there are no secondary SNPs flanking a query SNP, 86% (104/121) of the assays we designed converted. In contrast, just 65% (22/34) of the assays converted when a secondary SNP was present, and OLA oligos were designed to match only the major allele at that secondary SNP. It is of interest that the likelihood of an assay with a secondary SNP failing did not seem to depend on whether the secondary SNP was in the upstream or downstream oligo binding region, or on the distance of the secondary SNP from the query SNP. If we controlled for secondary SNPs by incorporating degenerate bases into the OLA oligos, then the success rate was equivalent (85%, 11/13) to that observed for query SNPs without secondary SNPs. Thus, our data suggest that if SNPs are identified via resequencing, employing degenerate bases in the OLA oligos can control for secondary SNPs.

For OLA assays that convert, but have an uncontrolled secondary SNP, the miscall rate can be appreciably higher than for sites without a secondary SNP. The OLA assay for site es09.C20633T in *Enhancer of split *did not control for a pair of secondary SNPs (one 8 base pairs (bp) upstream and one 9 bp downstream, both at a frequency 1/16 in the resequenced alleles) and converted to an apparently working assay. To check the accuracy of the OLA genotypes for es09.C20633T we sequenced 354 diploid individuals (GenBank accession numbers AY905900 to AY906258), and 3.1% (11/354) gave discordant genotypes. In each case a true C/T heterozygote was incorrectly called a T/T homozygote due to heterozygosity at a secondary SNP: in 10/11 individuals one of the previously identified segregating sites was to blame, while the remaining error was due to a previously unidentified SNP 1 bp downstream of the query SNP. Secondary SNPs may present a general problem for OLA-based genotyping methodologies, although their impact is dependent on the likelihood of there being a segregating site within the 16 base pairs upstream and downstream of the query SNP. Thus, for species with high levels of nucleotide diversity, such as *Drosophila*, the effect of secondary SNPs on OLA-based genotyping is expected to be more pronounced than for species with lower levels of diversity, such as humans.

### Hardy-Weinberg equilibrium

Adherence to Hardy-Weinberg equilibrium (HWE) is a common criterion with which to assess the quality of a genotyping assay, as a deviation can suggest incorrect genotype assignments [33]. However, selection, mutation or migration can also cause deviation from HWE, and the power to detect these processes increases with the sample size [34]. Of our 115 converting OLA assays with either no secondary SNPs or secondary SNPs controlled for via degenerate bases in the OLA oligos, 34 showed a deviation from HWE at *P *< 0.05. This is more than expected by chance, although the deviations from HWE were generally slight (the absolute mean disequilibrium for these 34 sites is *D *= 0.012). We hypothesized that the large panel size employed in our study (2,000 individuals) enabled detection of subtle violations of the HWE assumptions, which would not have been observed in a smaller panel. To test this hypothesis, we sampled 96 genotyped individuals at random from the population, and estimated the deviation from HWE for the same 115 SNPs. Over 1,000 sampled replicates, the average number of assays deviating from HWE was 8, similar to the 6 expected by chance alone.

### Genotype accuracy

To verify the accuracy of genotype calls from our OLA genotyping method, we performed a resequencing survey. Five regions from the *Enhancer of split *gene complex were selected in/near exons in an attempt to reduce the number of sequencing reads interrupted by heterozygous insertion/deletion polymorphisms, which are common in *Drosophila *noncoding DNA. The five sequenced regions collectively harbored 19 frequent (>5% minor allele frequency) genotyped SNPs (Table 1). Only one query SNP (es08.A16953T) exhibited a secondary SNP in the genotyping oligo binding region, which was controlled for via degenerate OLA genotyping oligos, and 13/19 showing no deviation from HWE at *P *< 0.05. We sequenced each of these regions in a sample of diploid individuals (GenBank accession numbers AY905719 to AY905899, AY906259 to AY906775) using the same PCR products used as template in the OLA reactions to provide a direct estimate of the accuracy of our genotyping assay. For four of the sequenced regions we sequenced 94 diploids (a single, arbitrarily selected 96-well plate of individuals, including two control samples), and for the fifth sequenced 375 diploids (a single, arbitrarily selected 384-well plate of individuals, including nine control samples), with no individual being sequenced for more than one region. Between 44 and 322 individuals gave genotypes for both the OLA and sequencing over the 19 SNPs (short sequencing reads, and failure to assign a genotype with the OLA assay is behind the difference between the number of sequenced individuals and the available data). The genotype intensity plots for the 19 tested SNPs are provided in Additional data file 12. From Table 1 it can be seen that the total accuracy rate is 1,715/1,721 (99.65%). This miscall rate of 0.35% is comparable to that of other technologies [14,16,17,29,35-38], and is only slightly higher than a value of 0.12% presented in Genissel *et al*. [39] for a comparison of just four SNPs genotyped by our OLA method and by allele-specific oligonucleotide (ASO) assays [24,40,41]. We note that 4/6 errors observed in the present study were due to individuals possessing a rare third allele at the query site that was not identified in the initial resequencing. Only methodologies that explicitly test for the presence of all four possible nucleotides at a query SNP, for example Hardenbol *et al*. [38,42], would correctly genotype these individuals. The remaining two errors we detected were from a single SNP, implying that the genotyping error rate varies among SNPs, and may be difficult to assess.

In the SNP genotyping literature, repeatability, or how often a technology gives concordant genotypes across replicates, is sometimes used as a surrogate for accuracy, or how often a technology yields the correct genotype. We suspect that the cases of incorrect genotype calls caused by uncontrolled secondary SNPs that we mention above are highly repeatable. Thus, for ligation-based genotyping of material not subject to resequencing multiple alleles, measures of repeatability will overestimate the genotyping accuracy for some SNPs.

### Conversion and call rate

We attempted to genotype 168 SNPs and biallelic insertion/deletion polymorphisms. If we ignore the 34 assays developed without regard to secondary SNPs in OLA genotyping oligo binding regions, 86% (115/134) of the assays convert. This conversion rate is particularly notable because it is derived from the actual production genotyping pipeline rather than independent proof-of-principal experiments. Furthermore, subsequent work has demonstrated very similar conversion rates for OLA genotyping assays conducted at 12- and 16-plex (data not shown). The call rate (that is, the number of individuals assigned a genotype) for the 115 converting assays here averages 93.9%, and we estimate the miscall rate to be <0.35%. Over the 115 converting assays, on average 1.1% of the individuals were assigned a genotype for only one of the two replicate spots on the membrane, and just 0.06% were assigned different genotypes for each replicate spot. Thus, for a very slight reduction in assay robustness, one could effectively double membrane density, and therefore throughput, by spotting samples only once.

### Comparison with existing methods

#### Technology

It has been pointed out by Syvänen [9,10] that while a plethora of SNP genotyping platforms exist, they are generally based on only a small number of basic reaction principles (for example, OLA [27], ASO [24,40,41], and single-base extension [43]), assay formats (for example, arrays, beads/microparticles, electrophoresis), and allele detection methods (for example, fluorescence, radiation, size separation, mass spectrometry). As such, most SNP genotyping platforms can be seen as modular, and the system we describe here is no exception: Following an initial, complexity-reducing PCR amplification, we genotype multiple SNPs in liquid-phase using OLA reactions, and subsequently detect SNP alleles by hybridizing radiolabeled probes to nylon membrane arrays.

Originally developed by Landegren *et al*. [27], many SNP genotyping methods have taken advantage of the high specificity and multiplexing capability of ligation-based genotyping [17,18,22,28,29,36,44-55]. A common way to distinguish the products of a multiplex genotyping reaction (not only OLA-based reactions) is to incorporate specific nucleotide sequences (variously called barcodes, addresses, zip-codes, stuffer sequences, or tags) into the allele-specific genotyping oligos [17,18,28,29,35,37,38,42,44,53-57]. In combination with fluorescent labeling of oligonucleotides, this procedure allows different SNPs, and alternative SNP alleles to be recognized. In the system we describe, alleles are detected by hybridizing radiolabeled oligonucleotide probes - complementary to the barcode sequences - to nylon membrane arrays of denatured, PCR amplified OLA products. This has the advantage of allowing a very large sample of individuals (up to 4,608) to be simultaneously genotyped for an intermediate number of SNPs (by probing multiple membranes). A reverse approach, pioneered by Gerry *et al*. [28], is to probe universal barcode, or tag arrays, with the genotyping reaction products, and discriminate alleles with fluorescent labels. The use of tag arrays has been employed in a variety of SNP genotyping technologies [16-18,28,35,37,38,42,54,55,57]. Given that the density of features on a tag array can be very high, methods that make use of them can genotype a very large number of SNPs simultaneously. However, because the number of individuals assayed is dependent on how many tag arrays can be examined, projects may be limited to hundreds, rather than thousands, of individuals. To increase the number of individuals assayed for a more modest number of SNPs, some researchers have had success using arrays-of-arrays [37,58]. Small tag arrays are printed in standard microtitre plate format, such that the contents of each well (a multiplexed genotyping reaction for a single individual) is hybridized to a separate array.

Array-based technologies are in widespread use. Arrays are used for applications as diverse as whole-genome expression profiling, polymorphism identification [59], and sequencing [60], and some of the companies providing ultra high-throughput genotyping solutions (for example, Illumina, Affymetrix) employ arrays. Nevertheless, SNP genotyping on arrays may not be an ideal solution for all researchers, particularly those with moderate genotyping requirements who may not wish to invest in array equipment. There are a variety of methods available that use the flexibility of ligation-based genotyping to generate sets of fluorescently labeled products of differing electophoretic mobility that can be resolved on an automated capillary sequencing instrument [22,29,44,46,48,52].

#### Cost

The full cost of any method is difficult to measure, and also may not translate well among institutions. We estimate that the cost in consumables (for example, oligonucleotides, reagents, plasticware, nylon membranes, and radiation/disposal costs), including the cost of failing assays, for the work presented in this study is less than $0.03/genotype. Across genotyping technologies, this is at the lower end of the cost per genotype scale. In common with every other genotyping method, some form of robotic liquid-handling system is required for our approach, as is a reasonable thermocycling capacity. Unlike some other methods however, the platform-specific requirements of the method we outline are few (membrane arraying robot, hybridization oven, phosphor imager, and phosphor screens), and we contend that much of this equipment is available to the majority of academic researchers, or in the case of the arraying robot, can be inexpensively built (Additional data file 6).

#### Applications

An ideal genotyping system, capable of genotyping millions of SNPs for thousands of individuals at low cost, does not exist. Therefore, the best genotyping method must be chosen on the basis of the specific requirements of the envisioned genotyping project, and the resources available. Our method adds to the diversity of the available technology, in particular because it fits into a multiplexing niche (high panel size, moderate number of SNPs) not well covered by existing technologies, and because of its open-source nature. Our method has been developed specifically for the high-depth association mapping applications we carry out in our laboratory (for example, Macdonald *et al*. [61]), and the method achieves cost-effectiveness in large part due to the very large panel sizes employed. Thus, the method is very unlikely to be suitable for projects involving thousands of SNPs in just a few hundred individuals, or for projects that do not involve a large fixed panel of individuals. Radioactive allele-detection also contributes to the low cost of the presented method. Such a detection strategy is clearly unwieldy in an ultra-high-throughput genome center. As such, we envisage our technology being employed in individual academic research laboratories where, given the widespread use of other radiation-based approaches, presumably utilizing radiation is not a barrier. The open-source nature of our platform, in contrast to similar commmercial genotyping systems (for example, Applied Biosystem's SNPlex), may also be attractive to some researchers, as it allows the technology to be altered to suit a specific need. The method we outline may fill a genotyping niche in an academic research environment where commercial solutions are unavailable, as is regularly the case for those working on the genetics of nonhuman systems.

## Conclusions

We describe a genotyping pipeline that uses a multiplexed OLA applied to PCR amplified DNA, followed by amplification of ligation products using common primers, and array-based detection. We tested 168 genotyping assays in parallel for a panel of 2,000 *D. melanogaster *individuals, and collected over a quarter of a million genotypes at a cost of less than $0.03/genotype. The assay conversion rate was 86%, and for converting assays 94% of the individuals were assigned a genotype with 99.65% accuracy, as determined by dideoxy sequencing. The methods we describe do not require a great deal of specialized equipment, and may be of great utility for carrying out high-power association mapping of candidate gene regions in individual laboratories. The methodology may help bridge the gap between highly multiplexed technologies capable of genotyping thousands of sites simultaneously, but which can be very costly for large samples of individuals, and methods that are easily extended to large populations, but can be difficult to multiplex beyond a small number of SNPs.

## Materials and methods

**Figure 1:**
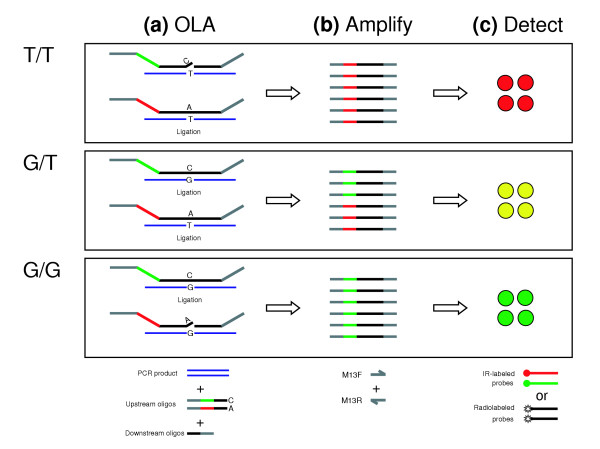
Principle of OLA-based SNP genotyping. **(a) **For each polymorphism, a set of three genotyping oligos are allowed to anneal to denatured PCR product (blue) in the presence of *Taq *DNA ligase. Ligation of up- and downstream oligos occurs only if there is a perfect match to template. Upstream oligos are color-coded gray (M13 forward amplification primer sequence), red/green (a pair of barcode sequences), and black (assay-specific sequence flanking the query SNP). The downstream oligo is 5'-phosphorylated, and color-coded gray (reverse complemented sequence of the M13 reverse amplification primer), and black (assay-specific flanking sequence). **(b) **Addition of common M13 primers (gray) allows amplification of all ligated products. **(c) **After arraying amplified OLA products, membranes are hybridized with probes complementary to the barcode sequences. Probes can be fluorescently labeled with infrared (IR) fluors and both alleles hybridized simultaneously, or radiolabeled and hybridized sequentially.

A broad outline of the method for a single SNP is shown in Figure 1, and complete step-by-step protocols are given in Additional data file 1.

### Genomic DNA and PCR amplification

Over 2,000 *Drosophila melanogaster *flies were collected from a single outbred population, and genomic DNA extracted from each using the PureGene cell and tissue DNA isolation kit (Gentra Systems Inc. Minneapolis, MN, USA). The DNA from each fly was diluted to 200 μl in 0.1 × TE (1 mM Tris-HCl pH 8.0, 0.1 mM EDTA), and 1 μl aliquoted directly into a series of 384-well plates and dried down. The resulting DNA panel consisted of six 384-well plates (including the 2,000 outbred individuals and a variety of controls), and each set of DNA was used as template in standard 5 μl PCR reactions. We amplified twelve 2 to 3 kb amplicons for the complete panel of *D. melanogaster *DNA: eleven amplicons were developed across the *Enhancer of split *locus, and a single amplicon was developed upstream of the *hairy *locus. Oligo sequences are listed in Additional data file 2.

### Genotyping oligos

We identified polymorphisms using an alignment of 16 resequenced alleles for the *Enhancer of split *locus (GenBank accession numbers AY779906 to AY779921; Additional data file 3) [31], and designed genotyping assays for 156 biallelic polymorphisms (both SNPs and simple insertion/deletion events). Also, an alignment of 10 alleles for the *hairy *locus (GenBank accession numbers AY055833 to AY055842) [32] was used to design genotyping assays for 12 SNPs upstream of the *hairy *gene. Genotyping oligo sequences are listed in Additional data file 2, and details of the polymorphisms are provided in Additional data file 4.

Three OLA genotyping oligos are required for each query SNP: two allele-specific upstream oligos (5'-M13F+C+BARCODE+U.FLANK-3') and a single common, 5'-phosphorylated downstream oligo (5'-D.FLANK+G+M13R.RC -3'). M13F (5'-GACGTTGTAAAACG-3') and M13R.RC (5'-CCTGTGTGAAATTG-3') are 14 nucleotide (nt) sequences matching the M13 forward amplification primer (5'-CCCAGTCACGACGTTGTAAAACG-3'), and the reverse complement of the M13 reverse primer (5'-AGCGGATAACAATTTCACACAGG-3'), respectively. A single 'C' ('G') nucleotide incorporated into the upstream (downstream) oligos after the M13 sequence may homogenize amplification across multiple products [44]. A 16 nt barcode sequence (Table 2) is incorporated into each upstream oligo and is used for SNP allele identification in a similar fashion to the design of genotyping primers described in Gerry *et al*. [28], and those used in various subsequent studies. We use a set of 16 pairs of barcodes, allowing up to 16-plex OLA reactions to be carried out, and 'recycle' barcodes to genotype multiple different SNPs across independent amplicons. The 16 nt sequence flanking each side of the query SNP is extracted from a multiple FASTA sequence alignment using our custom SNPatron perlscript (Additional data file 5).

Unmodified genotyping oligos were purchased at the lowest synthesis scale from Illumina Inc. (San Diego, CA, USA) and from Sigma-Genosys (St. Louis, MO, USA), and resuspended at a concentration of 100 μM in 1 × low EDTA TE (10 mM Tris-HCl pH 8.0, 0.1 mM EDTA). Downstream genotyping oligos were individually phosphorylated at the 5' end in 12.5 μl reactions containing 1 × T4 polynucleotide kinase buffer (New England Biolabs Inc., Ipswich, MA, USA), 1 mM ATP, 10 units T4 polynucleotide kinase (NEB), and 200 pmol oligo. These reactions were incubated for 60 minutes at 37°C and 20 minutes at 65°C. We found it difficult to reliably phosphorylate several oligonucleotides simultaneously (data not shown).

### OLA and OLA amplification reaction conditions

The OLA reactions are just 3 μl in volume, and contain 1 × OLA buffer (50 mM Tris-HCl pH 8.5, 50 mM KCl, 7.5 mM MgCl_2_, 1 mM NAD), 2.5 mM dithiothreitol, 1.6 units *Taq *(*Thermus aquaticus*) DNA ligase (NEB), and 0.03 pmol of each genotyping oligo. Each OLA reaction mix is spiked with 0.2 μl of PCR product using a HydraII 96-syringe pipetting unit (Matrix Technologies Corporation, Hudson, NH, USA). The small reaction sizes ensure that reagent costs are kept to a minimum. Ligation is performed using the following cycling profile: initial denaturation for 5 minutes at 95°C, followed by 3 cycles of 30 s at 95°C and 25 minutes at 45°C, followed by storage at 4°C. When perfectly matched up- and downstream oligos are juxtaposed to form a duplex with the amplified DNA they are ligated together (Figure 1a). The OLA is very efficient at discriminating between perfectly and imperfectly matched upstream oligonucleotides [27,44,62]. We genotyped 168 query polymorphisms using this approach; 160 of these were assayed in 20 8-plex OLA reactions using single 2 to 3 kb amplicons as template, while the remaining 8 were genotyped in a single 8-plex reaction using two pooled PCR amplicons as template.

Ligation products are PCR amplified using M13 forward and reverse primers matching the tails incorporated into the up- and downstream genotyping oligos (Figure 1b). To minimize plate handling, this is achieved by directly adding 12 μl post-OLA amplification cocktail directly to the OLA reactions. The amplification cocktail consists of 1 × amplification buffer (50 mM KCl, 0.1% Triton X-100), 50 μM each dNTP (NEB), *Taq *DNA polymerase, and 1 μM of the M13 forward and reverse amplification oligos. The ligation products are amplified using the following cycling profile: initial denaturation for 2 minutes at 94°C, followed by 32 cycles of 25 s at 94°C, 35 s at 58°C and 35 s at 72°C, followed by 2 minutes at 72°C, and storage at 4°C.

### Array-based allele detection

The 15 μl OLA amplification reactions are dried down at 65°C in a thermal cycler, resuspended in denaturing buffer (0.5 M NaOH, 1.5 M NaCl), heated for 15 minutes at 65°C and 5 minutes at 95°C, and immediately arrayed onto uncharged nylon membranes (Millipore Corporation, Billerica, NH, USA) without cleanup. Following UV cross-linking at 50 mJ, membranes are bathed in neutralization buffer (0.4 M Tris-HCl pH 7.4, 2× SSC) for 30 minutes, and stored at 4°C in neutralization buffer until required. Our home-built Cartesian arraying robot uses 384 solid pins (V & P Scientific Inc., San Diego, CA, USA), can be inexpensively constructed (Additional data file 6), and is controlled by our custom Arrayatron perlscript (Additional data file 7) from a regular PC. Our standard production macroarray membranes are 120 mm × 75 mm, and hold 4,608 features. Each sample was printed in duplicate, resulting in a set of 10 membranes holding SNPs incorporating barcode pairs 01 to 08, and a set of 11 membranes holding SNPs incorporating barcode pairs 09 to 16. Each set of membranes were combined in single hybridization tubes, and pre-hybridized for 3 hours (overnight for first use of membranes) at 42°C in 5 ml hybridization buffer (0.525 M sodium phosphate buffer pH 7.2, 7% SDS, 1 mM EDTA, 10 mg/ml bovine serum albumin) containing 0.1 mg/ml denatured sonicated herring sperm DNA. Following pre-hybridization, the membranes were hybridized for 4 hours at 42°C in 5 ml hybridization buffer with 0.1 mg/ml denatured sonicated herring sperm DNA and a [γ-^33^P]ATP end-labeled oligonucleotide probe (complementary to the barcode sequence; Table 2). The 10 μl end-labeling reaction contains 1 × T4 polynucleotide kinase buffer (NEB), 10 units T4 polynucleotide kinase (NEB), 1 μM oligo, and 2 μCi/μl [γ-^33^P]ATP (PerkinElmer Life and Analytical Sciences Inc., Boston, MA, USA), and is incubated for 40 minutes at 37°C and 15 minutes at 80°C. After hybridization, the membranes are washed five times for 20 minutes at 40°C in 50 ml washing buffer (5 × SSPE, 0.1% SDS), and exposed against phosphor screens (Figure 1c). After scanning, membranes are stripped for 15 minutes at 80°C in 50 ml stripping buffer (0.1% SDS), and stored at 4°C in neutralization buffer until re-probing.

In concert with recycling barcodes across different SNPs, hybridizing multiple membranes allows simultaneous scoring of many SNPs. Radioactive detection is cost-effective, robust, and does not require a great deal of equipment (for example, hybridization oven, phosphor imager) not already available to many investigators. We have found, however, that the same arrays simultaneously probed with two infrared-labeled probes (IR-700 and IR-800) and detected using an Odyssey imaging system (Li-Cor Inc., Lincoln, NE, USA) yield equivalent genotypes. This non-radioactive detection system has several advantages and may prove a worthwhile extension to our method.

### Genotype scoring

**Figure 2:**
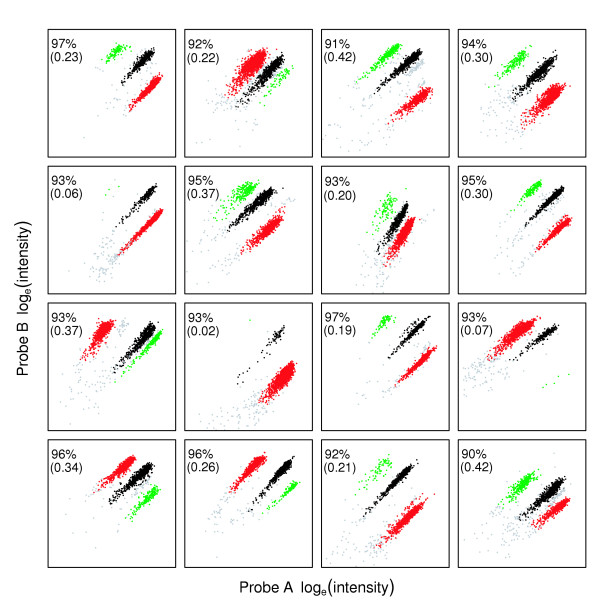
A representative set of SNP genotyping assays. Each of the 16 panels represents a single SNP selected using a random number generator from the set of 115 converting OLA genotyping assays (from top to bottom, and left to right: es13.C29977T, es13.A30471C, es03.G6361A, es08.A16882G, es08.A17666C, es09.T20794C, es19.T43316G, es02.T2815G, es20.in47414del, es03.G5471A, es02.C3366T, es08.C16678T, es13.A29956G, es17.A40101T, es08.C16807T, es03.T6871G). Each intensity plot displays approximately 2,000 points, representing single *D. melanogaster *individuals, color-coded to reflect the assigned genotype (red, major allele homozygote; black, heterozygote; green, minor allele homozygote; gray, no assigned genotype). The legend for each panel is the percentage of individuals assigned a genotype, and (in parentheses) the frequency of the minor allele.

A major advantage of array-based genotyping over gel- or capillary-based approaches is the relative ease of automated data extraction. We use ArrayVision (v8.0, Imaging Research Inc., St. Catharines, Ontario, Canada) to quantify the intensity of each spot from the images obtained by scanning the phosphor screen. The resulting intensity data are passed to a custom script (Additional data file 8) written in the freely available statistical programming language R [63]. This script reads in the intensity data for each allele of a SNP, allows the user to define spots representing the three genotype classes (*AA*, *Aa*, and *aa*), then implements a likelihood function to assign a genotype, or a no-call, to each individual (see Genissel *et al*. [39] for a detailed description of the likelihood function). Because each sample is printed in duplicate on the membranes, the genotype assigned to an individual is a consensus of the genotypes applied to the replicate pair of spots: if both spots give the same genotype, or if only one spot yields a genotype (and the other a no-call), that genotype is assigned, but if the spots give different genotypes, the individual is assigned a no-call. Our genotype calling procedure, while requiring some user intervention, allows rapid, accurate genotype calling. Figure 2 highlights the data quality for a random set of 16 converting OLA genotyping assays. Assays are deemed to convert if the intensity plots show three clear genotype clusters (or two in the case of rare SNPs).

## Additional data files

The following additional files are available with the online version of this article. Additional data file 1 is a PDF providing full step-by-step protocols for the described SNP genotyping platform. Additional data file 2 is a spreadsheet giving all of the oligonucleotide sequences used for PCR, sequencing and genotyping. Additional data file 3 holds the alignment of the 16 *D. melanogaster *alleles sequenced for the *Enhancer of split *gene region. Additional data file 4 is a spreadsheet providing details of the 168 polymorphisms assayed in this study. Additional data file 5 is the SNPatron perlscript, used to extract the sequence flanking all SNPs and polymorphic insertion/deletion events from a set of aligned sequences. Additional data file 6 is a PDF describing the construction of our arraying robot. Additional data file 7 presents the Arrayatron perlscript used to control the arraying robot. Additional data file 8 gives the script used to call genotypes, which is written in the statistical programming language R. The background-subtracted array intensity data for each allele from each genotyped site are provided in Additional data files 9 (replicate spot 1) and 10 (replicate spot 2), and the called genotypes are given in Additional data file 11. Additional data file 12 plots the intensity data for the entire panel of individuals for the 19 SNPs used in the genotype-validation test, with the tested individuals color-coded by the genotype assigned.

## Supplementary Material

Additional data file 1 Detailed protocols for the presented SNP genotyping platform. Click here for file

Additional data file 2 Oligonucleotide sequences for PCR, OLA genotyping, and sequencing. Click here for file

Additional data file 3 Alignment of 16 resequenced *Drosophila melanogaster *alleles for the complete *Enhancer of split *gene complex. Click here for file

Additional data file 4 Details of the 168 SNPs and insertion/deletion polymorphisms genotyped. Click here for file

Additional data file 5 Passing a sequence alignment in FASTA format to the script will return tables of the SNPs and insertion/deletion polymorphisms present in the alignment, and a consensus sequence. Click here for file

Additional data file 6 This includes a parts list, diagrams, and photographs of the system. Click here for file

Additional data file 7 This script allows the user to control the movement of our custom-built Cartesian arraying robot with a regular PC. Click here for file

Additional data file 8 This script is written in the freely available statistical programming language R, and allows the user to take the intensity data for each allele of each spot from the hybridized membranes, and assign genotypes to each spot. Click here for file

Additional data file 9 Each row represents a *D. melanogaster *individual, or a blank. The first column is the name of the individual (or blank), the second column identifies the replicate spot (spot 1), and the remaining columns hold the intensity data, with alleles from the same polymorphism in consecutive columns. The column names for the intensity data are constructed from the amplicon within which the site resides, its position (in base pairs) in a sequence alignment, the SNP allele, and the barcode marking the allele. Click here for file

Additional data file 10 Each row represents a *D. melanogaster *individual, or a blank. The first column is the name of the individual (or blank), the second column identifies the replicate spot (spot 2), and the remaining columns hold the intensity data, with alleles from the same polymorphism in consecutive columns. The column names for the intensity data are constructed from the amplicon within which the site resides, its position (in base pairs) in a sequence alignment, the SNP allele, and the barcode marking the allele. Click here for file

Additional data file 11 The first column is the individual name, and the remaining columns hold genotype data (NA, no genotype assigned; 0, minor allele homozygote; 1, heterozygote; 2, major allele homozygote). The column names for the genotype data are constructed from the amplicon within which the site resides, the major allele, the position (in base pairs) of the site in a sequence alignment, and the minor allele. Click here for file

Additional data file 12 Each plot displays approximately 2,000 points, representing single *D. melanogaster *individuals. The points representing individuals assigned genotypes by an OLA assay and by sequencing are colored and large, while the remaining individuals are shown as smaller gray points. Red, major allele homozygote in both OLA and sequencing; black, heterozygote in both OLA and sequencing; green, minor allele homozygote in both OLA and sequencing; yellow, OLA and sequencing yield different genotypes. Click here for file

## Figures and Tables

**Table 1:** Genotype accuracy

SNP	Number of OLA and sequence data points*	Identical data points	% Identical
es02.C3366T	79	79	100
es02.A3435G^†^	89	89	100
es02.A3707C	86	86	100
es08.A16615C^†^	75	75	100
es08.C16678T	77	77	100
es08.C16807T	75	75	100
es08.A16882G	71	71	100
es08.A16953T	44	44	100
es12.G27418A	66	66	100
es12.T27548A	81	81	100
es12.T27764C^†^	76	76	100
es12.T27800C	69	67	97.10
es13.A29775G	86	86	100
es13.A29833C^†^	90	87^‡^	96.67
es13.A29956G	85	85	100
es13.C29977T	89	89	100
es13.C30040T	79	79	100
es13.C30152T^†^	82	81^‡^	98.78
es15.G35074A^†^	322	322	100
Total	1,721	1,715	99.65

*The number of individuals assigned a genotype both in the OLA genotyping assay and by direct sequencing of the PCR product used in the assay. ^†^SNP genotypes are out of Hardy-Weinberg equilibrium at *P *< 0.05. ^‡^These differences between the genotypes given by OLA and sequencing are due to rare third alleles at the query SNP that were not seen in the initial resequencing.

**Table 2:** Probes and barcode sequences

Probe number	Probe/barcode *a*	Probe/barcode *b*
01	ATATTCTGAGACACGCCGCG	ATACGCGATGGGATCAGACT
02	ATGCGACTCTTGACGAACGT	TTCGAGCGTCTGGCACACTT
03	GTCACTCGTGTCCAGGATGT	TATCGCGTGTCAGTGCTTGT
04	GATACCGGACCATGTTTCGC	GATGTTCGTCCATGCGACCT
05	TGATCCGCGTCGATGCTCTT	GCAGTCACGTTCTCGAATCG
06	TTTAGCCGGATCACCGTGTG	ATATGTGCAGAACCCGCGAC
07	AGAGAGACGTTGCCCAAGTC	GATGCGATACCCTGCGATCT
08	ATTTAGCGTGCAGCCGACCT	ATGCGTGGTGTCCGATCATA
09	TAAGGGTTACGAACATCGCC	TGGACTCTCATAACGGCGTC
10	GCAGCTCGTCACAGGTATTG	TACCGGATTACAGCTCGTGG
11	AGCTAATGTCGAGTCACGCT	TCTACACGAGAACGAGGCAC
12	AGCGCGACGTTGATCCAGAT	AATGAACGAGACCGCGTGAC
13	TCGGACTCGTGACGCTATTT	ATGAGAGTTCGATGACCTGT
14	ACGCACTGACGATCATTCGG	TTCGACCCGGACGACTGTAT
15	TATAGCCGTGAACCCGATGC	TAAAGCACAGTCCGTAATCT
16	ATCATGTCCCAAGCGCGGTA	AAGCCGATGTCGATCTACCT

All 20 nucleotide probe sequences are given in the 5' to 3' direction. The 16 nucleotide barcode sequences incorporated into the upstream oligonucleotide ligation assay oligos are the reverse complement of the underlined portion.

## References

[B1] Carlson CS, Eberle MA, Kruglyak L, Nickerson DA (2004). Mapping complex disease loci in whole-genome association studies.. Nature.

[B2] Risch N, Merikangas K (1996). The future of genetic studies of complex human diseases.. Science.

[B3] Kruglyak L (1999). Prospects of whole-genome linkage disequilibrium mapping of common disease genes.. Nat Genet.

[B4] Long AD, Langley CH (1999). The power of association studies to detect the contribution of candidate genetic loci to variation in complex traits.. Genome Res.

[B5] Kruglyak L, Nickerson DA (2001). Variation is the spice of life.. Nat Genet.

[B6] Altshuler D, Hirschhorn JN, Klannemark M, Lindgren CM, Vohl MC, Nemesh J, Lane CR, Schaffner SF, Bolk S, Brewer C (2000). The common PPARγPro12Ala polymorphism is associated with decreased risk of type 2 diabetes.. Nat Genet.

[B7] Lohmueller KE, Pearce CL, Pike M, Lander ES, Hirschhorn JN (2003). Meta-analysis of genetic association studies supports a contribution of common variants to susceptibility to common disease.. Nat Genet.

[B8] Kwok PY (2001). Methods for genotyping single nucleotide polymorphisms.. Annu Rev Genomics Hum Genet.

[B9] Syvänen AC (2001). Accessing genetic variation: genotyping single nucleotide polymorphisms.. Nat Rev Genet.

[B10] Syvänen AC (2005). Toward genome-wide SNP genotyping.. Nat Genet.

[B11] Hinds DA, Stuve LL, Nilsen GB, Halperin E, Eskin E, Ballinger DG, Frazer KA, Cox DR (2005). Whole-genome patterns of common DNA variation in three human populations.. Science.

[B12] Fodor SP, Read JL, Pirrung MC, Stryer L, Lu AT, Solas D (1991). Light-directed, spatially addressable parallel chemical synthesis.. Science.

[B13] Pease AC, Solas D, Sullivan EJ, Cronin MT, Holmes CP, Fodor SP (1994). Light-generated oligonucleotide arrays for rapid DNA sequence analysis.. Proc Natl Acad Sci USA.

[B14] Matsuzaki H, Loi H, Dong S, Tsai YY, Fang J, Law J, Di X, Liu WM, Yang G, Liu G (2004). Parallel genotyping of over 10,000 SNPs using a one-primer assay on a high-density oligonucleotide array.. Genome Res.

[B15] Matsuzaki H, Dong S, Loi H, Di X, Liu G, Hubbell E, Law J, Berntsen T, Chadha M, Hui H (2004). Genotyping over 100,000 SNPs on a pair of oligonucleotide arrays.. Nat Methods.

[B16] Oliphant A, Barker DL, Stuelpnagel JR, Chee MS (2002). BeadArray technology: enabling an accurate, cost-effective approach to high-throughput genotyping.. Biotechniques.

[B17] Fan JB, Oliphant A, Shen R, Kermani BG, Garcia F, Gunderson KL, Hansen M, Steemers F, Butler SL, Deloukas P (2003). Highly parallel SNP genotyping.. Cold Spring Harbor Symp Quant Biol.

[B18] Shen R, Fan JB, Campbell D, Chang W, Chen J, Doucet D, Yeakley J, Bibikova M, Wickham Garcia E, McBride C (2005). High-throughput SNP genotyping on universal bead arrays.. Mutat Res.

[B19] Ronaghi M, Uhlen M, Nyren P (1998). A sequencing method based on real-time pyrophosphate.. Science.

[B20] Alderborn A, Kristofferson A, Hammerling U (2000). Determination of single-nucleotide polymorphisms by real-time pyrophosphate DNA sequencing.. Genome Res.

[B21] Livak KJ (1999). Allelic discrimination using fluorogenic probes and the 5' nuclease assay.. Genet Anal.

[B22] De La Vega FM, Lazaruk KD, Rhodes MD, Wenz MH (2005). Assessment of two flexible and compatible SNP genotyping platforms: TaqMan SNP genotyping assays and the SNPlex genotyping system.. Mutat Res.

[B23] Chen X, Levine L, Kwok PY (1999). Fluorescence polarization in homogeneous nucleic acid analysis.. Genome Res.

[B24] Saiki RK, Bugawan TL, Horn GT, Mullis KB, Erlich HA (1986). Analysis of enzymatically amplified β-globin and HLA-DQα DNA with allele-specific oligonucleotide probes.. Nature.

[B25] Mullis KB, Faloona FA (1987). Specific synthesis of DNA *in vitro* via a polymerase-catalyzed chain reaction.. Methods Enzymol.

[B26] Southern EM (1975). Detection of specific sequences among DNA fragments separated by gel electrophoresis.. J Mol Biol.

[B27] Landegren U, Kaiser R, Sanders J, Hood L (1988). A ligase-mediated gene detection technique.. Science.

[B28] Gerry NP, Witowski NE, Day J, Hammer RP, Barany G, Barany F (1999). Universal DNA microarray method for multiplex detection of low abundance point mutations.. J Mol Biol.

[B29] van Eijk MJ, Broekhof JL, van der Poel HJ, Hogers RC, Schneiders H, Kamerbeek J, Verstege E, van Aart JW, Geerlings H, Buntjer JB (2004). SNPWave: a flexible multiplexed SNP genotyping technology.. Nucleic Acids Res.

[B30] The Patrick Brown Laboratory Guide to Microarraying. http://cmgm.stanford.edu/pbrown/mguide/index.html.

[B31] Macdonald SJ, Long AD (2005). Identifying signatures of selection at the *Enhancer of split *neurogenic gene complex in *Drosophila*.. Mol Biol Evol.

[B32] Robin C, Lyman RF, Long AD, Langley CH, Mackay TF (2002). *hairy *: a quantitative trait locus for *Drosophila* sensory bristle number.. Genetics.

[B33] Hosking L, Lumsden S, Lewis K, Yeo A, McCarthy L, Bansal A, Riley J, Purvis I, Xu CF (2004). Detection of genotyping errors by Hardy-Weinberg equilibrium testing.. Eur J Hum Genet.

[B34] Weir BS (1996). Genetic Data Analysis II.

[B35] Hirschhorn JN, Sklar P, Lindblad-Toh K, Lim YM, Ruiz-Gutierrez M, Bolk S, Langhorst B, Schaffner S, Winchester E, Lander ES (2000). SBE-TAGS: an array-based method for efficient single-nucleotide polymorphism genotyping.. Proc Natl Acad Sci USA.

[B36] Faruqi AF, Hosono S, Driscoll MD, Dean FB, Alsmadi O, Bandaru R, Kumar G, Grimwade B, Zong Q, Sun Z (2001). High-throughput genotyping of single nucleotide polymorphisms with rolling circle amplification.. BMC Genomics.

[B37] Bell PA, Chaturvedi S, Gelfand CA, Huang CY, Kochersperger M, Kopla R, Modica F, Pohl M, Varde S, Zhao R (2002). SNPstream UHT: ultra-high throughput SNP genotyping for pharmacogenomics and drug discovery.. Biotechniques.

[B38] Hardenbol P, Banér J, Jain M, Nilsson M, Namsaraev EA, Karlin-Neumann GA, Fakhrai-Rad H, Ronaghi M, Willis TD, Landegren U, Davis RW (2003). Multiplexed genotyping with sequence-tagged molecular inversion probes.. Nat Biotechnol.

[B39] Genissel A, Pastinen T, Dowell A, Mackay TF, Long AD (2004). No evidence for an association between common nonsynonymous polymorphisms in *Delta *and bristle number variation in natural and laboratory populations of *Drosophila melanogaster*.. Genetics.

[B40] Wallace RB, Shaffer J, Murphy RF, Bonner J, Hirose T, Itakura K (1979). Hybridization of synthetic oligodeoxyribonucleotides to Φχ174 DNA: the effect of single base pair mismatch.. Nucleic Acids Res.

[B41] Conner BJ, Reyes AA, Morin C, Itakura K, Teplitz RL, Wallace RB (1983). Detection of sickle cell β^*S*^-globin allele by hybridization with synthetic oligonucleotides.. Proc Natl Acad Sci USA.

[B42] Hardenbol P, Yu F, Belmont J, MacKenzie J, Bruckner C, Brundage T, Boudreau A, Chow S, Eberle J, Erbilgin A (2005). Highly multiplexed molecular inversion probe genotyping. Over 10,000 targeted SNPs genotyped in a single tube assay.. Genome Res.

[B43] Syvänen AC, Aalto-Setala K, Harju L, Kontula K, Söderlund H (1990). A primer-guided nucleotide incorporation assay in the genotyping of apolipoprotein E.. Genomics.

[B44] Schouten JP, McElgunn CJ, Waaijer R, Zwijnenburg D, Diepvens F, Pals G (2002). Relative quantification of 40 nucleic acid sequences by multiplex ligation-dependent probe amplification.. Nucleic Acids Res.

[B45] Nickerson DA, Kaiser R, Lappin S, Stewart J, Hood L, Landegren U (1990). Automated DNA diagnostics using an ELISA-based oligonucleotide ligation assay.. Proc Natl Acad Sci USA.

[B46] Grossman PD, Bloch W, Brinson E, Chang CC, Eggerding FA, Fung S, Iovannisci DM, Woo S, Winn-Deen ES (1994). High-density multiplex detection of nucleic acid sequences: oligonucleotide ligation assay and sequence-coded separation.. Nucleic Acids Res.

[B47] Samiotaki M, Kwiatkowski M, Parik J, Landegren U (1994). Dual-color detection of DNA sequence variants by ligase-mediated analysis.. Genomics.

[B48] Eggerding FA (1995). A one-step coupled amplification and oligonucleotide ligation procedure for multiplex genetic typing.. PCR Methods Appl.

[B49] Delahunty C, Ankener W, Deng Q, Eng J, Nickerson DA (1996). Testing the feasibility of DNA typing for human identification by PCR and an oligonucleotide ligation assay.. Am J Hum Genet.

[B50] Tobe VO, Taylor SL, Nickerson DA (1996). Single-well genotyping of diallelic sequence variations by a two-color ELISA-based oligonucleotide ligation assay.. Nucleic Acids Res.

[B51] Nilsson M, Krejci K, Koch J, Kwiatkowski M, Gustavsson P, Landegren U (1997). Padlock probes reveal single-nucleotide differences, parent of origin and *in situ *distribution of centromeric sequences in human chromosomes 13 and 21.. Nat Genet.

[B52] Favis R, Day JP, Gerry NP, Phelan C, Narod S, Barany F (2000). Universal DNA array detection of small insertions and deletions in *BRCA1* and *BRCA2*.. Nat Biotechnol.

[B53] Iannone MA, Taylor JD, Chen J, Li MS, Rivers P, Slentz-Kesler KA, Weiner MP (2000). Multiplexed single nucleotide polymorphism genotyping by oligonucleotide ligation and flow cytometry.. Cytometry.

[B54] Busti E, Bordoni R, Castiglioni B, Monciardini P, Sosio M, Donadio S, Consolandi C, Rossi Bernardi L, Battaglia C, De Bellis G (2002). Bacterial discrimination by means of a universal array approach mediated by LDR (ligase detection reaction).. BMC Microbiol.

[B55] Banér J, Isaksson A, Waldenström E, Jarvius J, Landegren U, Nilsson M (2003). Parallel gene analysis with allele-specific padlock probes and tag microarrays.. Nucleic Acids Res.

[B56] Chen J, Iannone MA, Li MS, Taylor JD, Rivers P, Nelsen AJ, Slentz-Kesler KA, Roses A, Weiner MP (2000). A microsphere-based assay for multiplexed single nucleotide polymorphism analysis using single base chain extension.. Genome Res.

[B57] Fan JB, Chen X, Halushka MK, Berno A, Huang X, Ryder T, Lipshutz RJ, Lockhart DJ, Chakravarti A (2000). Parallel genotyping of human SNPs using generic high-density oligonucleotide tag arrays.. Genome Res.

[B58] Pastinen T, Raitio M, Lindroos K, Tainola P, Peltonen L, Syvänen AC (2000). A system for specific, high-throughput genotyping by allele-specific primer extension on microarrays.. Genome Res.

[B59] Borevitz JO, Liang D, Plouffe D, Chang HS, Zhu T, Weigel D, Berry CC, Winzeler E, Chory J (2003). Large-scale identification of single-feature polymorphisms in complex genomes.. Genome Res.

[B60] Zwick ME, Mcafee F, Cutler DJ, Read TD, Ravel J, Bowman GR, Galloway DR, Mateczun A (2005). Microarray-based resequencing of multiple *Bacillus anthracis *isolates.. Genome Biol.

[B61] Macdonald SJ, Pastinen T, Long AD (2005). The effect of polymorphisms in the *Enhancer of split *gene complex on bristle number variation in a large wild-caught cohort of *Drosophila melanogaster*.. Genetics.

[B62] Luo J, Bergstrom DE, Barany F (1996). Improving the fidelity of *Thermus thermophilus *DNA ligase.. Nucleic Acids Res.

[B63] The R Project for Statistical Computing. http://www.R-project.org.

