# Supplementary material for: A low-cost open-source SNP genotyping platform for association mapping applications
Source: Genome Biol. 2005 Dec 2;6(12):R105. doi: 10.1186/gb-2005-6-12-r105 (PMC1414086; doi:10.1186/gb-2005-6-12-r105)
Supplement: Additional data file 1 — Detailed protocols for the presented SNP genotyping platform. [file gb-2005-6-12-r105-S1.pdf]

S. J. Macdonald, UC Irvine, July 2005

SINGLE-FLY DNA EXTRACTION

(reagents in bold from Gentra Systems DNA isolation kit)

Cell lysis

1. Add 100 µl ice-cold **cell lysis solution** to a 1.5 ml tube containing a single fly
2. Homogenize fly for ~5 sec with a motor-driven pestle
3. Incubate at 65°C for 15 min
4. Cool sample to room temperature

RNase treatment

5. Add 2 µl of a 1:4 dilution of **RNase A solution** (≡ 1mg/ml) to cell lysate
6. Mix sample by inverting tube 25 times, and incubate at 37°C for 40 min
7. Cool sample to room temperature

Protein precipitation

8. Add 33 µl **protein precipitation solution** to the RNase-treated cell lysate
9. Vortex on high speed for 10 sec, and place sample on ice for 5 min
10. Centrifuge at 14,000 rpm for 3 min

DNA precipitation

11. Move supernatent to a new 1.5 ml eppendorf containing 100 µl isopropanol
12. Mix by inverting tube gently 50 times
13. Centrifuge at 14,000 rpm for 5 min, and gently pour off supernatent
14. Add 100 µl 70% ethanol, and invert tube a few times to wash pellet
15. Centrifuge at 14,000 rpm for 1 min, and remove supernatent with pipette
16. Invert tube on absorbant paper and leave to air dry for 20 min

DNA resuspension

17. Add 20 µl sterile-filtered 1X TE (pH 8.0) to tube
18. Resuspend overnight at room temperature
19. Add 180 µl ddH_20 to tube. 0.5-1 µl of this DNA is suitable for a standard 5 µl PCR reaction

S. J. Macdonald, UC Irvine, July 2005

RE-ARRAY DNA

(DNA from tubes to plates)

1. Move diluted DNA into 96-well microtitre plates
   → For long-term –20°C/–80°C storage of 96-well DNA stock plates, it is advisable to use plates with caps rather than adhesive seals

2. Aliquot 1 μl diluted DNA from 96-well plates to 384-well plates using the HydraII liquid handler

3. Spin the 384-well plates, dry samples in a thermocycler at 65°C for 5 min, seal, and store at –20°C/–80°C until PCR

PCR

(2-3kb PCR with ExTaq DNA polymerase)

1. Design PCR oligos for desired region using the web-based *Primer3* program
   → http://frodo.wi.mit.edu/cgi-bin/primer3/primer3_www.cgi
   → change "Product Size Ranges" box to appropriate values, but otherwise retain defaults

2. Make up the following PCR cocktail (2,350 reactions is sufficient for six 384-well plates, aliquoted using the Hydra from a V-groove reagent reservoir):

	1 × 5 µl	2,350 × 5 µl
ddH_2O	3.825	8988.75
ExTaqBuffer (10X)	0.5	1175
dNTPs (2.5 mM)	0.4	940
ExTaq (5U/µl)	0.025	58.75
F.oligo (5 µM)	0.125	293.75
R.oligo (5 µM)	0.125	293.75

3. Add 5 µl of cocktail to each well of the 384-well plates, seal, and briefly spin down

4. Use a thermocycling profile similar to:

STEP.1	95°C	5 min
STEP.2	95°C	30 sec
(35 cycles)	55°C	30 sec
	72°C	2 min 30 sec
STEP.3	72°C	4 min
STEP.4	4°C	hold

S. J. Macdonald, UC Irvine, July 2005

Genotyping Oligos

(design oligos, create oligo mixes for 8-plex OLA)

Finding polymorphisms: `SNPatron`

1. Generate a standard multiple FASTA file of aligned sequences using the normal IUB nucleotide codes (gaps should be identified with hyphens "–"):

```
> gene sequence 1
AGCTTGCTGTATTTCATCGGTTTCCATGGGCCTATCGATCCGGCATGCCATGCATG
CGCGACTCGCGC---GCGCTACGCGCGCATTAGGGGGGGGAT
> gene sequence 2
AGCTAGCAGTATTTCATCGGATTCCA------TATCGATCCGGCTTGCCATGCTTG
CGCGACTCGCGGTACGCCCTACGCGCGCATTAGGGGGGGGAT
> gene sequence 3
AGCTAGCAGTATTTCATCGGATTCCATGGGCCTATCGATCCGGCTTGCCATGCTTG
CGCGACTCGCGG---GCCCTACGCGCGCATTAGGGGGGGGAT
```

2. Pass file through custom SNPatron perlscript to extract all biallelic SNPs and insertion/deletion polymorphisms, and the 16 bases flanking each side of the polymorphic site, which are used as the sequence-specific portion of the genotyping oligos
   → the software will fail to run if the aligned sequences are not of identical length

Designing genotyping oligos

3. Once a set of SNPs/InDels have been selected for genotyping, two upstream allele-specific oligos, and a single common downstream oligo, are designed as follows:

   UPSTREAM "a" 5′- M13F + C + BARCODE "a" + U.FLANK allele 1 -3′

   UPSTREAM "b" 5′- M13F + C + BARCODE "b" + U.FLANK allele 2 -3′

   DOWNSTREAM 5′- D.FLANK + G + M13R.RC -3′

   Notes:

   * M13 sequence is 14 nt. The sequence in the downstream oligo is the reverse complement of M13R. The sequences allow ligated oligos to be PCR amplified

     M13F 5′-GAC GTT GTA AAA CG-3′
     M13R.RC 5′-CCT GTG TGA AAT TG-3′

   * BARCODEs "a" and "b" are 16 nt barcode sequences permitting allele discrimination during hybridization. Barcodes are used in pairs, and 16 pairs are

available. Within any given mix of genotyping oligos, it is essential that each assayed polymorphism uses a different barcode pair

* U.FLANK and D.FLANK are 16 nt and sequence-specific to the regions flanking the polymorphic site, and are provided by the `SNPatron` perlscript. The U.FLANK regions of the pair of upstream oligos differ only by the polymorphic nucleotide at the 3′ end

* The 'C' or 'G' nucleotide adjacent to the M13 sequence ensures that multiple ligated products of different sequence are evenly amplified (for details see Schouten *et al.*, 2002. Relative quantification of 40 nucleic acid sequences by multiplex ligation-dependent probe amplification. Nucleic Acids Research 30 e57)

Genotyping oligo mixes

4. Oligos can be purchased at the lowest possible synthesis scale (often 25 nmol), with standard desalting purification, and should be resuspended at 100 μM in sterile-filtered 1X low-EDTA TE
5. *Upstream Oligo Mix:* Add 2 μl of each of the 16 upstream oligos (at 100 μM) to 168 μl ddH_2O ⇒ 200 μl mix at 1 μM with respect to each upstream oligo
6. *Downstream Oligo Mix:* Each of the 8 downstream oligos must be phosphorylated independently (phosphorylation *en masse* fails to phosphorylate all oligos to the same extent):

Reagents:

	1 × 12.5 μl
ddH_2O	8.125
T4 Polynucleotide Kinase Buffer (10X)	1.25
ATP (100 mM)	0.125
T4 Polynucleotide Kinase (10U/μl)	1
Downstream oligo (100 μM)	2

Incubation:

STEP.1	37°C 1 hr
STEP.2	65°C 20 min
STEP.3	4°C hold

Mix:

Add 12 μl of phosphorylation mix for each of the 8 oligos to 96 μl ddH_2O ⇒ 192 μl at 1 μM with respect to each downstream oligo

Oligonucleotide Ligation Assay

(8-plex OLA)

1. Make up the following OLA cocktail (2,405 reactions is sufficient for six 384-well plates, aliquoted using the Hydra):

	1 × 3 µl	2,405 × 3 µl
ddH_2O	2.3	5531.5
OLA Buffer (10X) [a]	0.3	721.5
DTT (25 mM) [b]	0.3	721.5
Taq DNA Ligase (40U/µl)	0.04	96.2
Upstream oligo mix [c]	0.03	72.15
Downstream oligo mix [c]	0.03	72.15

2. Aliquot 3 µl cocktail to each well of the 384-well plates, and keep plates on ice

3. Use the Hydra to spike the OLA cocktail with 0.2 µl of gDNA PCR, seal the plates, and briefly spin down

4. Cycle using the following temperature profile:

STEP.1	95°C	5 min
STEP.2	95°C	30 sec
(3 cycles)	45°C	25 min
STEP.3	4°C	hold

Notes:

[a] 10X OLA buffer = 500 mM Tris-HCl pH 8.5, 75 mM $MgCl_2$, 10 mM NAD, 500 mM KCl. Since OLA buffer contains NAD, buffer should be kept at -20°C to maintain NAD activity. Long-term storage of 10X OLA buffer should be at -80°C

[b] DTT is sensitive to freeze/thaw cycles, which should be kept to a minimum

[c] See previous section “Genotyping Oligos”

OLA Amplification

(ligation-dependent amplification)

1. Make up the following cocktail to amplify the ligated products from the OLA reactions (2,400 reactions is sufficient for six 384-well plates, aliquoted using the Hydra):

	1 × 12 µl	2,400 × 12 µl
ddH_2O	10.056	24134.4
OLAamp Buffer (10X) [a]	1.2	2880
dNTPs (25 mM)	0.024	57.6
Taq	0.24	576
M13F.BRL (50 µM) [b]	0.24	576
M13R.BRL (50 µM) [c]	0.24	576

2. Add 12 µl of cocktail to each well of the 384-well plates holding the 3 µl OLA reaction products, seal the plates, and briefly spin down

3. Amplify products using the following thermocycling profile:

STEP.1	94°C	2 min
STEP.2	94°C	25 sec
(32 cycles)	58°C	35 sec
	72°C	35 sec
STEP.3	72°C	2 min
STEP.4	4°C	hold

Notes:

[a] 10X OLA amplification buffer = 500 mM KCl, 1% Triton X-100

[b] M13F.BRL 5′-CCC AGT CAC GAC GTT GTA AAA CG-3′

[c] M13R.BRL 5′-AGC GGA TAA CAA TTT CAC ACA GG-3′

[b/c] If fewer than 8 polymorphisms are to be tested, primer concentrations should be reduced appropriately

Arraying

(array amplified OLA products)

Prepare OLA products for printing

1. Dry 15 μl OLA amplification reactions at 65°C in thermocycler for ~1 hr

2. Add 5 μl of sterile-filtered denaturing buffer to each well of 384-well plate

3. Resuspend/denature the samples in thermocycler:

STEP.1	65°C	15 min
STEP.2	95°C	5 min

Prepare membranes

4. Cut membranes to 75 mm height × 120 mm width – gloves must be worn throughout

5. Tape membranes to deck of printing robot, ensuring they are flat against the surface

Printing

6. Slide 384-well plates, holding denatured OLA amplification products, into printing robot plate holders

7. Fill the four printing robot pin-washing stations: (1) 2% bleach, (2) ddH_2O, (3) ddH_2O, (4) 95% EtOH, set up appropriate printing protocol, and start robot using `Arrayatron` perlscript

Prepare membranes for hybridization

8. Once print run is complete, wait 10 min for printed samples to dry, label membranes using a pencil, and carefully remove from printing robot deck
9. UV cross-link samples onto the membranes at 50 mJ
10. Gently shake membranes in a bath of neutralization buffer at 40 rpm for at least 30 min
11. Membranes can then be stored in neutralization buffer at 4°C until hybridization

Hybridization

(probe membranes with radiolabeled oligos)

Pre-hybridization

1. Add membrane(s) to a hybridization tube
   → membranes can be stacked, provided they are slightly offset, and two stacks of 6 (= 12 total) membranes can be effectively hybridized/washed in a single hybridization tube

2. Add 5 ml of pre-heated (42°C) hybridization buffer, and 50 µl of denatured (96°C for 5 min) sonicated herring sperm DNA (at 10mg/ml)

3. Spin tube in hybridization oven at 42°C and 4 rpm:
   (a) overnight for first probing of membrane
   (b) 3 hr for all subsequent probings

Hybridization

4. Prepare radiolabeled oligonucleotide probe by end-labeling oligo with γ^{33}ATP:

   Reagents:

	1 × 10 µl
ddH_2O	5
T4 Polynucleotide Kinase Buffer (10X)	1
Probe oligo (10 µM) [a]	1
T4 Polynucleotide Kinase (10U/µl)	1
ATP-γ^{33}P (10µCi/µl)	2

   Incubation:

STEP.1	37°C 40 min
STEP.2	80°C 15 min

   Notes:

   [a] Probe stock concentration = 100 µM in 1X low-EDTA TE, diluted 1/10 with ddH_2O ⇒10 µM solution.

5. Briefly centrifuge labeling reaction mix, inject radiolabeled probe directly into hybridization tube, and spin at 4 rpm and 42°C for 4 hr
   → if membranes were pre-hybridized overnight, replace the hybridization buffer and herring sperm DNA

Washing

6. Empty hybridization buffer/radiolabeled probe to waste

7. Briefly rinse inside of hybridization tube with a small quantity of washing buffer, pre-heated at 40°C, and discard to waste

8. Add ~50 ml of 40°C washing buffer to hybridization tube, and let tube spin at 4 rpm for 20 min at 40°C

9. Discard washing buffer to waste, and repeat step (8) four times for a total of five wash cycles

10. Remove membranes from hybridization tube and rinse briefly in a bath of 40°C washing buffer

Expose membrane to phosphor screen

11. Place washed membranes on surface of the phosphor screen cassette, cover with a layer of transparent food wrap, and remove all bubbles by wiping surface with a Kimwipe

12. Close cassette with phosphor screen, and leave in dark for 3-4 nights

13. Scan phosphor screen using a phosphor imager
    → image pixel size should be at most 88 microns

Membrane stripping

14. Move probed/exposed membranes from cassette to bath of neutralization buffer, then add membranes to hybridization tube with ~50ml of stripping buffer pre-heated to 80°C
    → membranes are added to neutralization buffer before stripping to keep them wet – drying membranes before stripping risks permanently fixing probe to membrane

15. Spin tube in hybridization oven for 15 min at 80°C and 4 rpm

16. Discard buffer to waste, remove membranes from tube and either store in neutralization buffer at 4°C, or briefly rinse in neutralization buffer before re-probing

17. Clean hybridization tube and screw-cap with water to remove residual salts/radiation. Clear phosphor screen on light box for 12 min to eliminate previous image

Data Analysis

(data acquisition and genotype calling)

Generating Data Files

1. Using the *ArrayVision* software (v8.0, Imaging Research Inc., www.imagingresearch.com) extract the intensity data from all of the printed spots for each membrane

2. *ArrayVision* can be set to output a tab-delimited *.txt datafile holding three columns:
   (a) spot position (row/column), OR an identifying spot name
   (b) the average intensity data of the spot
   (c) the background-subtracted intensity data of the spot

3. After probing for both alleles of a SNP (or SNPs), create a tab-delimited *.txt file holding the background-subtracted intensity data for each allele of a SNP in adjacent columns. A typical data file may look like:

spot.position	blanks	plate	snp01_A	snp01_G	snp02_T	snp02_C
R1–C1	1	1	500.543	123.678	325.287	851.112
R1–C2	1	2	1023.478	1974.500	6593.789	1025.895
R1–C3	2	3	478.390	935.987	678.097	842.976

   * spot.position, blanks, and plate are various indicator columns – any number of these are possible, BUT for subsequent calling using our R script the columns “plate” and “blanks” are essential
   → none of these indicator columns should contain non-standard symbols or whitespaces
   * the four data columns represent two SNPs (snp01 and snp02), with a column for each allele of the two SNPs (*i.e.* snp01 is an AG polymorphism, and snp02 is a TC polymorphism)

4. This file is now ready to be run though our custom SNP genotype calling code using R

Genotype Calling

5. The code is written in the statistical programming language R (www.R-project.org), and a description of the calling routine is described in Genissel *et al.* 2004. No Evidence for an Association Between Common Nonsynonymous Polymorphisms in *Delta* and Bristle Number Variation in Natural and Laboratory Populations of *Drosophila melanogaster*. Genetics 166: 291-306

Buffers

1X TE

10 mM Tris-Cl pH 8.0
1 mM EDTA pH 8.0

1X low-EDTA TE

10 mM Tris-Cl pH 8.0
0.1 mM EDTA pH 8.0

Denaturing Buffer

0.5 M NaOH
1.5 M NaCl

For 1 litre:

20 g NaOH
87.66 g NaCl
→ make up to 1 litre with ddH_2O

Neutralization Buffer

0.4 M Tris-HCl pH 7.4
2X SSC

For 1 litre:

400 ml 1M Tris-HCl pH 7.4
100 ml 20X SSC
→ make up to 1 litre with ddH_2O

Reagents:

1 litre of 1 M Tris-HCl pH 7.4:
121.1 g Tris-HCl
69-70 ml HCl to pH
→ to 1 litre with ddH_2O

1 litre of 20X SSC pH 7.0:
175.3 g NaCl
88.2 g $Na_3C_6H_5O_7 \bullet 2H_2O$
drop 10 M NaOH to pH
→ to 1 litre with ddH_2O

Hybridization Buffer

0.525 M sodium phosphate (NaPi) pH 7.2
7% SDS
1 mM EDTA pH 8.0
10mg/ml bovine serum albumin (BSA)

For 40 ml:

21 ml 1 M NaPi pH 7.2
14 ml 20% SDS
5 ml 8 mM EDTA pH 8.0
0.4 g BSA

Reagents:

1 litre of 1 M NaPi pH 7.2:
134 g $Na_2HPO_4 \bullet 7H_2O$
4 ml H_3PO_4
→ to 1 litre with ddH_2O

Washing Buffer	5X SSPE<br>0.1% SDS		
For 1 litre:	250 ml 20X SSPE pH 7.4<br>745 ml ddH_2O<br>5 ml 20% SDS		
Reagents:	1 litre of 20X SSPE pH 7.4:	175.3 g NaCl<br>27.6 g NaH_2PO_4•H_2O<br>7.4 g EDTA<br>~20ml 10 M NaOH to pH<br>→ to 1 litre with ddH_2O	
Stripping Buffer	0.1% SDS		
For 1 litre:	995 ml ddH_2O<br>5 ml 20% SDS		

Consumables

Item	Catalog Number	Website	Phone (US)
Puregene Cell and Tissue DNA Isolation Kit (1 kit)	D5000A	www.gentra.com	1-888-476-5283
Cycleplate-384 DW PCR Plates (10 plates)	1047-00-0	www.abgene.com	1-800-445-2812
ExTaq DNA Polymerase (12 × 250 Units)	TAKRR_001C	www.takaramirusbio.com	1-888-251-6618
Taq DNA Ligase (10,000 Units)	M0208L	www.neb.com	1-800-632-5227
T4 Polynucleotide Kinase (500 Units)	M0201S	www.neb.com	1-800-632-5227
dNTPs (25μmol each)	N0446S	www.neb.com	1-800-632-5227
MicroAmp 384-well PCR Plate Adhesive Seals (100 seals)	4306311	www.appliedbiosystems.com	1-800-345-5224
Easytides Adenosine 5'-Triphosphate $\gamma^{33}P$ (250μCi)	NEG602H250UC	las.perkinelmer.com	1-800-551-2121
Sonicated Herring Sperm DNA (100mg, 10μg/μl)	D1815	www.promega.com	1-800-356-9526
Disposable V-groove Automation Reservoir (96 channel, 20 units)	1064-05-8	www.matrixtechcorp.com	1-800-345-0206
96-well Clear PCR Plates (50 plates)	3430	www.clpdirect.com	1-800-456-7741
Immobilon Nylon Uncharged Transfer Membrane (0.45μm, 30cm × 3m roll)	INYU00010	www.millipore.com	1-800-645-5476
Filter Paper, 3MM grade (100 sheets, 35cm × 45cm)	3030-392	www.whatman.com	1-800-441-6555

Equipment

Hydra II
Matrix Technologies, www.matrixtechcorp.com, 1-800-345-0206

109611	Hydra II 96 base unit with Dureflex needles (including ControlMate), 100μl syringes
10961XY	2-postion X/Y plate stage (includes laptop PC and auto syringe wash module)

Thermocyclers
Applied Biosystems, www.appliedbiosystems.com, 1-800-345-5224

N805-0002	Dual 384-well GeneAmp PCR system 9700

Hybridization Oven/Tubes
SciGene, www.scigene.com, 1-800-342-2119

1040-50-1	Model 2000 hybridization incubator
1040-01-0	Hybridization tube, 35mm × 300mm, with screw-cap

UV Cross-linker
Bio-Rad, www.biorad.com, 1-800-424-6723

NA	GS Gene Linker UV chamber, 254nm

Phosphor Imaging
Amersham Biosciences, www.amershambiosciences.com, 1-800-526-3593

63-34-82	Storage Phosphor Screen (mounted, screen and cassette), 35cm × 43cm
	PhosphorImager 445 SI
