# Supplementary material for: A low-cost open-source SNP genotyping platform for association mapping applications
Source: Genome Biol. 2005 Dec 2;6(12):R105. doi: 10.1186/gb-2005-6-12-r105 (PMC1414086; doi:10.1186/gb-2005-6-12-r105)

Equipment

Velmex
www.velmex.com
1-800-642-6446

See attached parts list

VP Scientific
www.vp-scientific.com
1-800-455-0644

FP1	0.457mm solid tube pins
AFIX384FP1	Floating frame fixture for 384 0.457mm diameter FP1 pins
VP581	PCR plate flattener for warped plates

Hardware Store

Particle board	2 sheets (185cm × 62cm × 16mm)<br>[fixed together to make up robot deck]
Timber	4 pieces (25mm × 25mm × 62cm each)<br>[support the deck]
Screws	12 screws, 3 per support (44mm long)<br>[to fix the 2 sheets of particle board to timber supports]
	18 screws, 3 per plate holder (12mm long)<br>[to fix plate holders to deck]
Bolts	28 bolts (44mm long)<br>[to mount the BiSlide rails to the deck – mounting fixtures provided by Velmex, Inc.]

Laboratory Supplier

Tip-box lids	4 lids<br>[for the 4 wash stations]
Lab tape	used to temporarily fix nylon membranes to the deck of the robot

VELMEX, INC.
7550 State Routes 5 & 20 Bloomfield, NY 14469-9389
Tel: 585-657-6151 Fax: 585-657-6153 E-Mail: dave@velmex.com

Quote Number: DE212016 Rev 0
Quote Create Date: 12/10/02
Current Revision Date: 12/10/02

(Refer to Quotation No. when placing order)

Total Pages Sent: 1

Ref: Verbal RFQ

Description: Velmex Positioning System with Motor and Controller
3 axis BISLIDE system
Travel: 50 x 15 x 5 inches; Lea screws: X&Y - 0.400 inch/rev, Z - 0.100 inch/rev;
Resolution: approx 0.005 inch (X&Y), 0.001 inch (Z); Max Speeds: X - 50 mm/sec, Y - 100 mm/sec, Z - 38 mm/sec
Payload: <5 lb

Item	Qty	Cat No	Description	Unit Price	Total
1	1	MN10-0500-E04-31	BiSlide, t=50 inch, 0.4 in/rev, Limits, NEMA34 Mt	$1,316.00	$1,316.00
2	1	4-9834	Motor, Stepper Type 34T2, SS, M092-FD-447	$190.00	$190.00
3	1	MN10-0150-E04-31	BiSlide, t=15 inch, 0.4 in/rev, Limits, NEMA34 Mt	$790.00	$790.00
4	1	4-9832	Motor, Stepper Type 34T1, SS, M091-FD-454	$155.00	$155.00
5	1	MN10-0050-E01-21	BiSlide, t=5 inch, 0.1 in/rev, Limits, NEMA23 Mt	$715.00	$715.00
6	1	4-9827	Motor, Stepper Type 23T2, DS, M062-LS-551E	$165.00	$165.00
7	1	4-940	Damper Vexta D6CL-6.3	$88.00	$88.00
8	1	4-2103	VXM-3 Prog Controller for 3 motors w/power supply	$1,535.00	$1,535.00
9	2	MC-2	Mounting Cleat (1.42 inch pattern) (BiS to BiS)	$5.00	$10.00
10	14	MC-3	Mounting Cleat (2.00 inch pattern) (Bench mount)	$5.00	$70.00
11	7	MB-1	Bolt - 1/4-20x3/4 Socket head cap (10 pack)	$2.90	$20.30

Note: All required cables for motors, limit switches & communication included
Shipping Charge Estimate: $72.00 via UPS-GND (approx 7 days)

**Subtotal:**	$5,054.30
**Volume Discount:**	
**Net Total:**	$5,054.30

This system is a complete positioning system with motors and controller capable of motion over an area of 50 x 15 x 5 inches. Minimal setup is required for turnkey operation. Setup consists of mounting X axis to bench with MC-3 cleats provided, mounting Y axis to X axis with MC-2 cleats provided, mounting Z axis to Y axis with 4 bolts and connecting all cables.
Our COSMOS Visual Basic software is included for quick start up and initial setting of parameters.
A windows based PC is required for programming purposes.

Shipping: 1 to 2 weeks ARO -- Pre-Pay and Add
Terms: Net 30
FOB: Bloomfield, NY 14469
Valid for: 30 Days

Regards,

[signature]

David W. Engdahl
Technical Sales - Motorized Assemblies

Cartesian arraying robot: PLAN KEY

- = deck
- = deck support
- 1 = WASH station (2% bleach)
- 2 = WASH station (ddH_2O)
- 3 = WASH station (ddH_2O)
- 4 = WASH station (95% ethanol)
- = 384-well PLATE HOLDER
  [V&P Scientific Cat. VP581]
- = Nylon membrane (120mm X 75mm)
- x z = axis (x, y or z)
- = BiSlide rail [Velmex, Inc.]
- = direction of movement
- PINS = Floating frame fixture for 384 pins
  [V&P Scientific Cat. AFIX384FP1]
- = Custom pin-tool mounting arm
  [see attached plan]

Cartesian arraying robot: PLAN VIEW

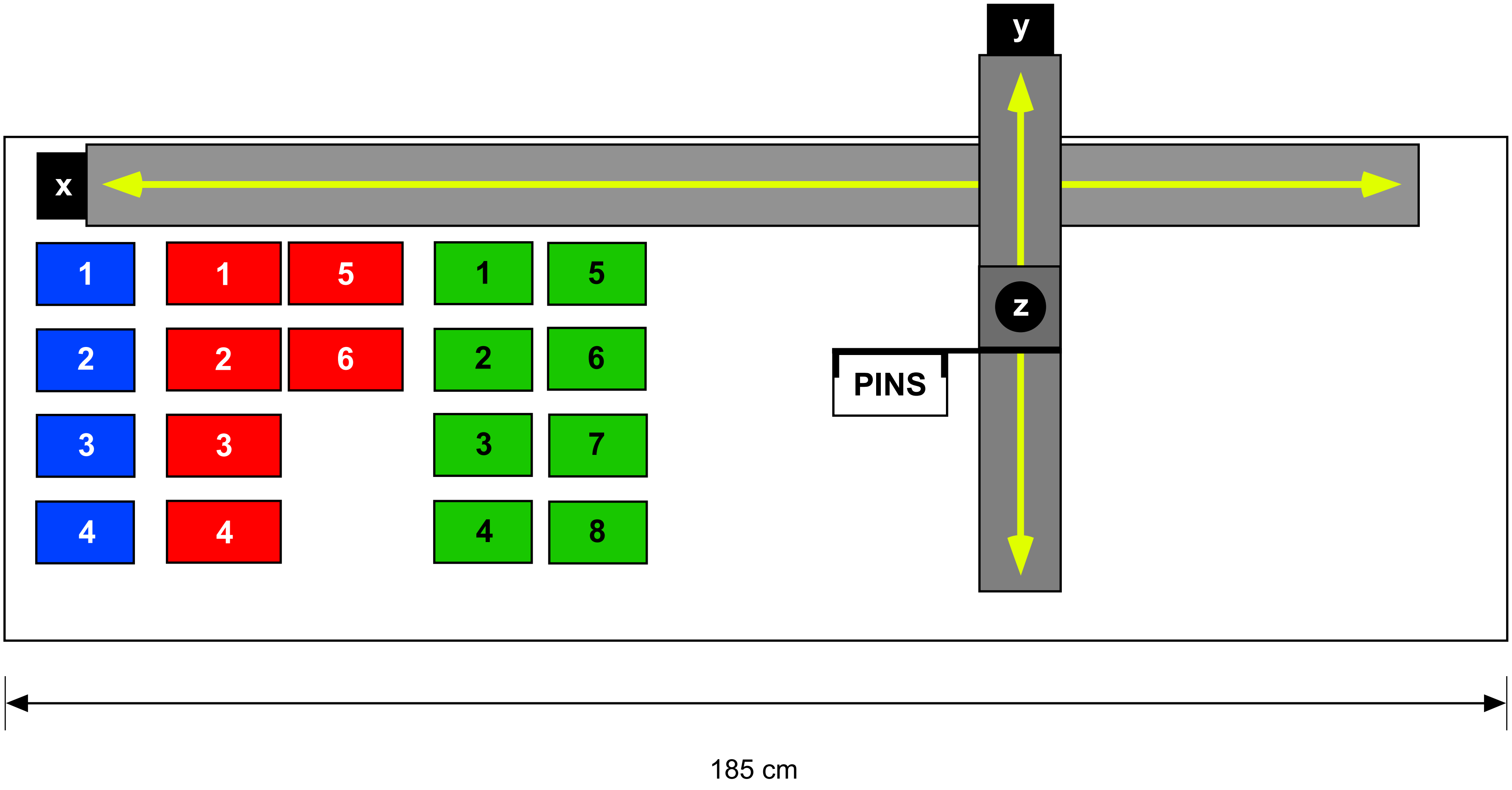

Cartesian arraying robot: FRONT VIEW

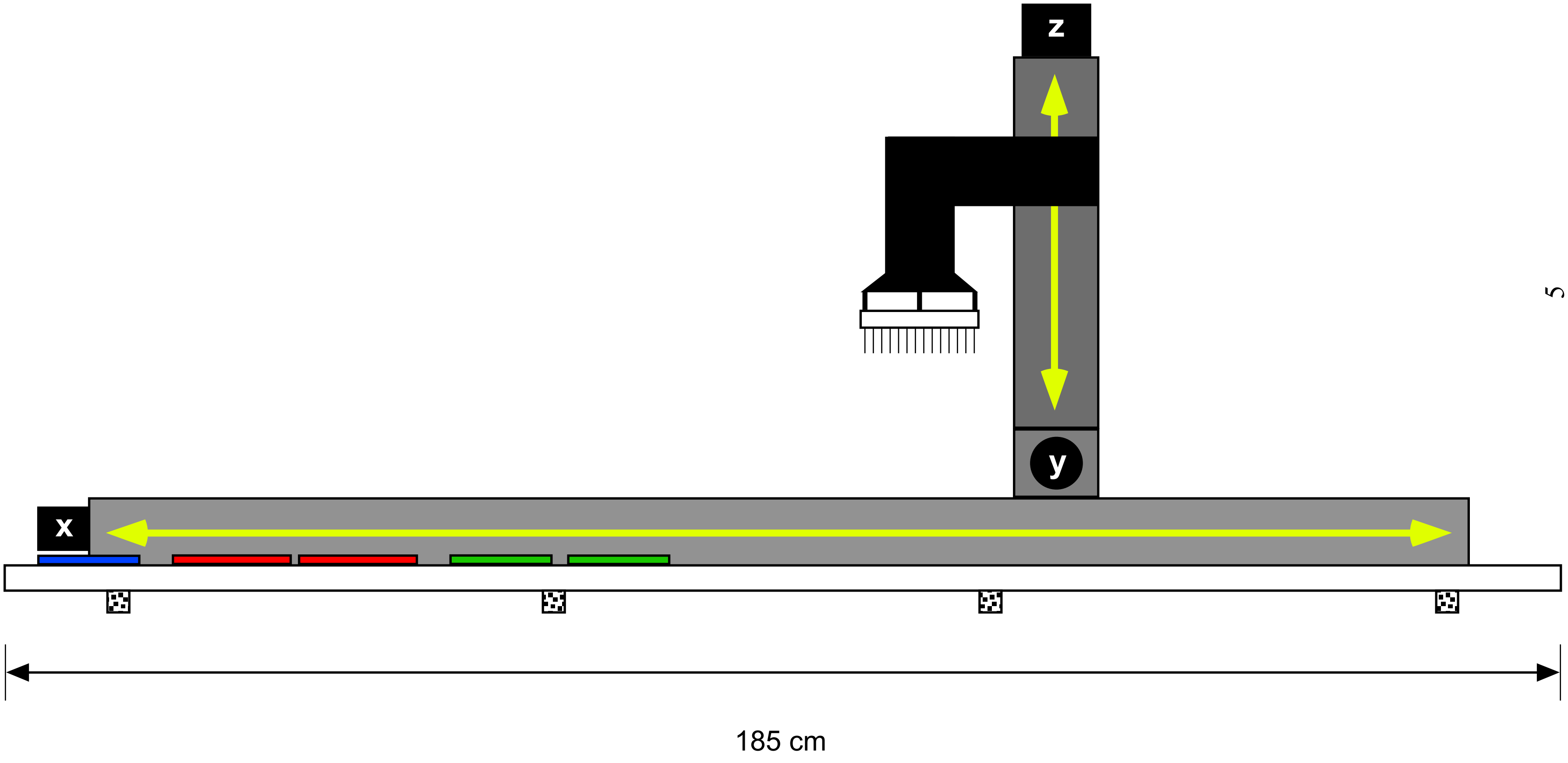

Cartesian arraying robot: SIDE VIEW

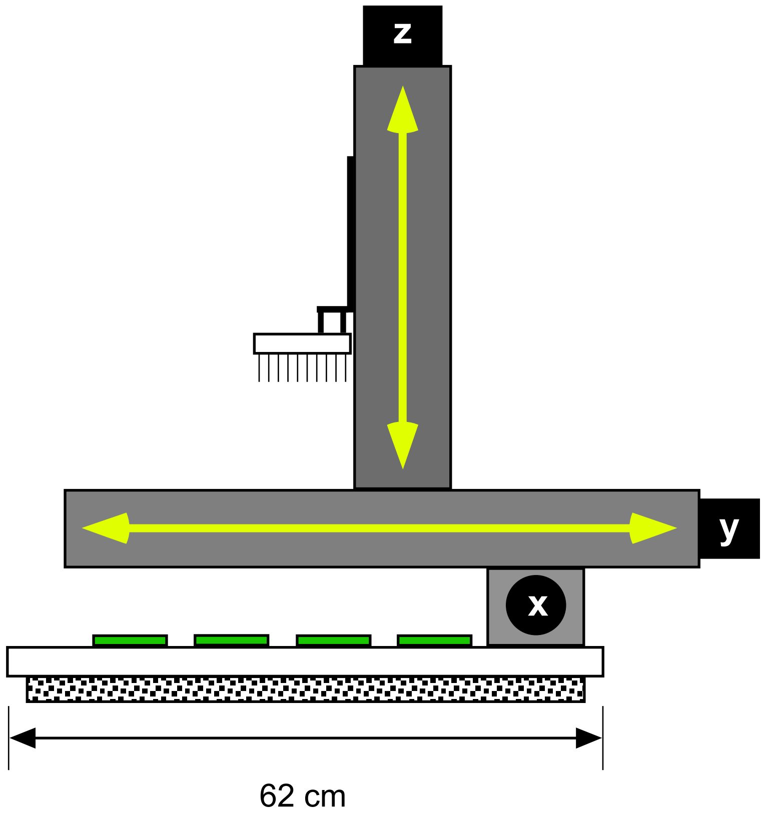

Custom Pin-tool Mounting Arm

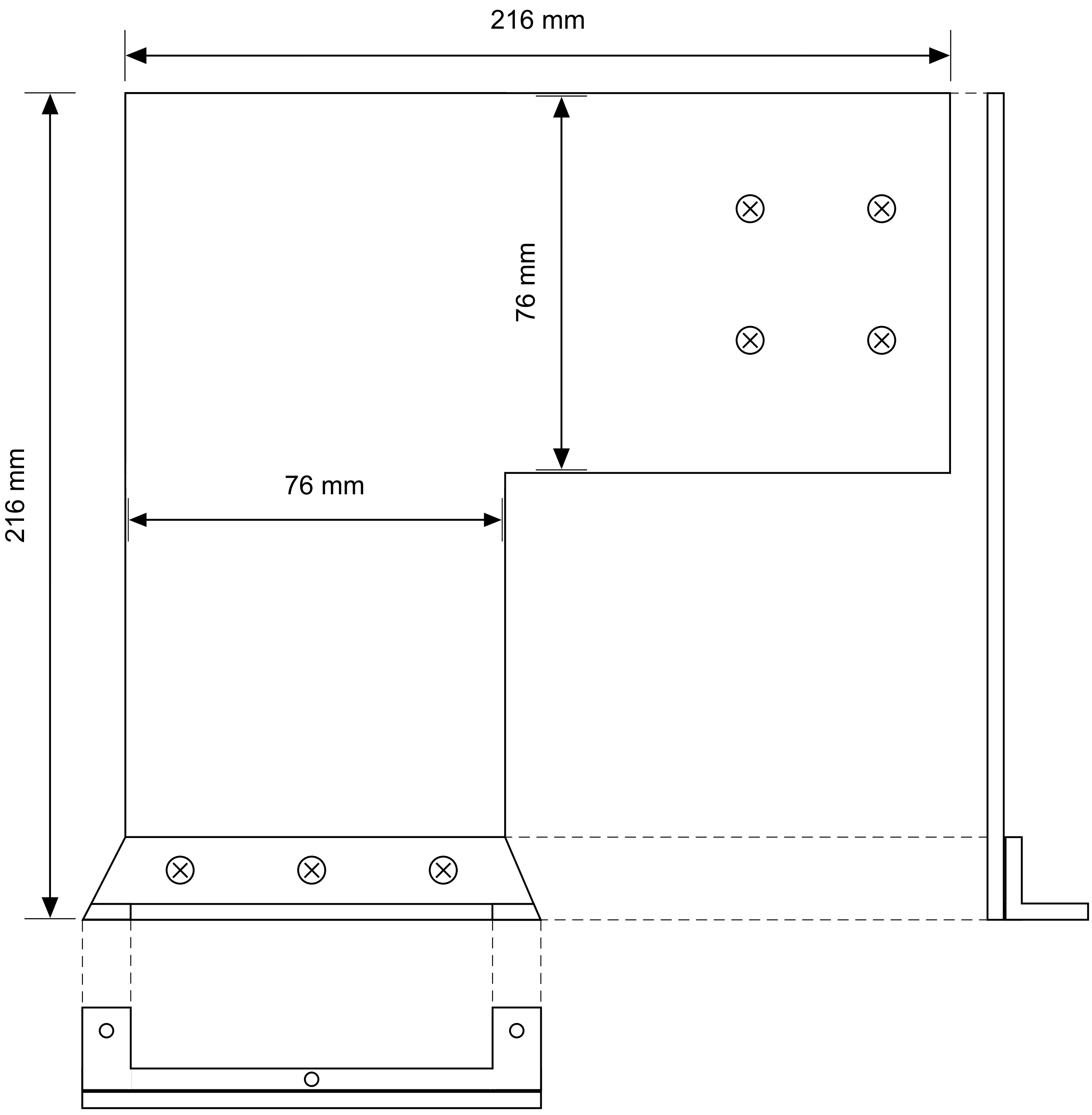

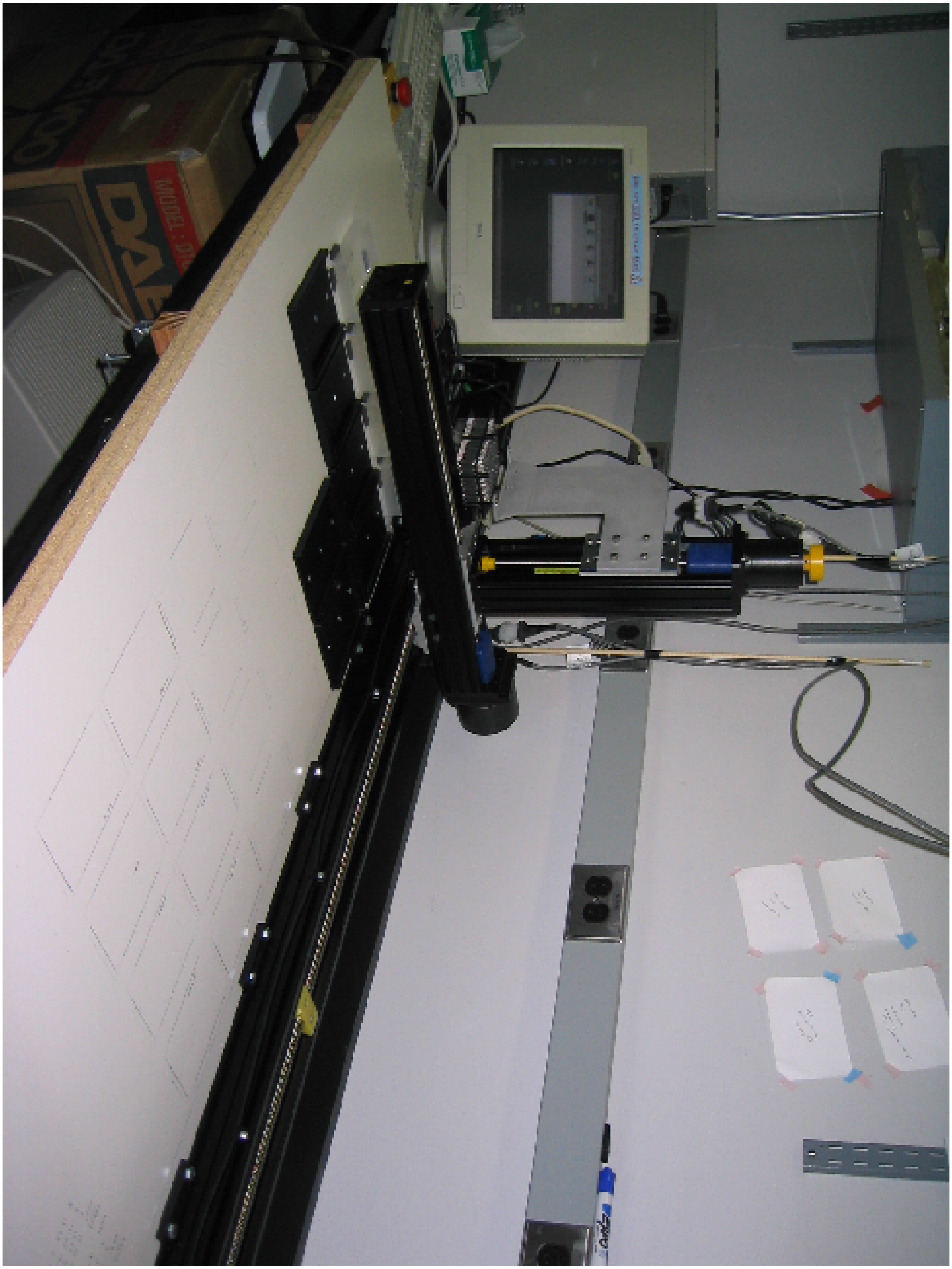

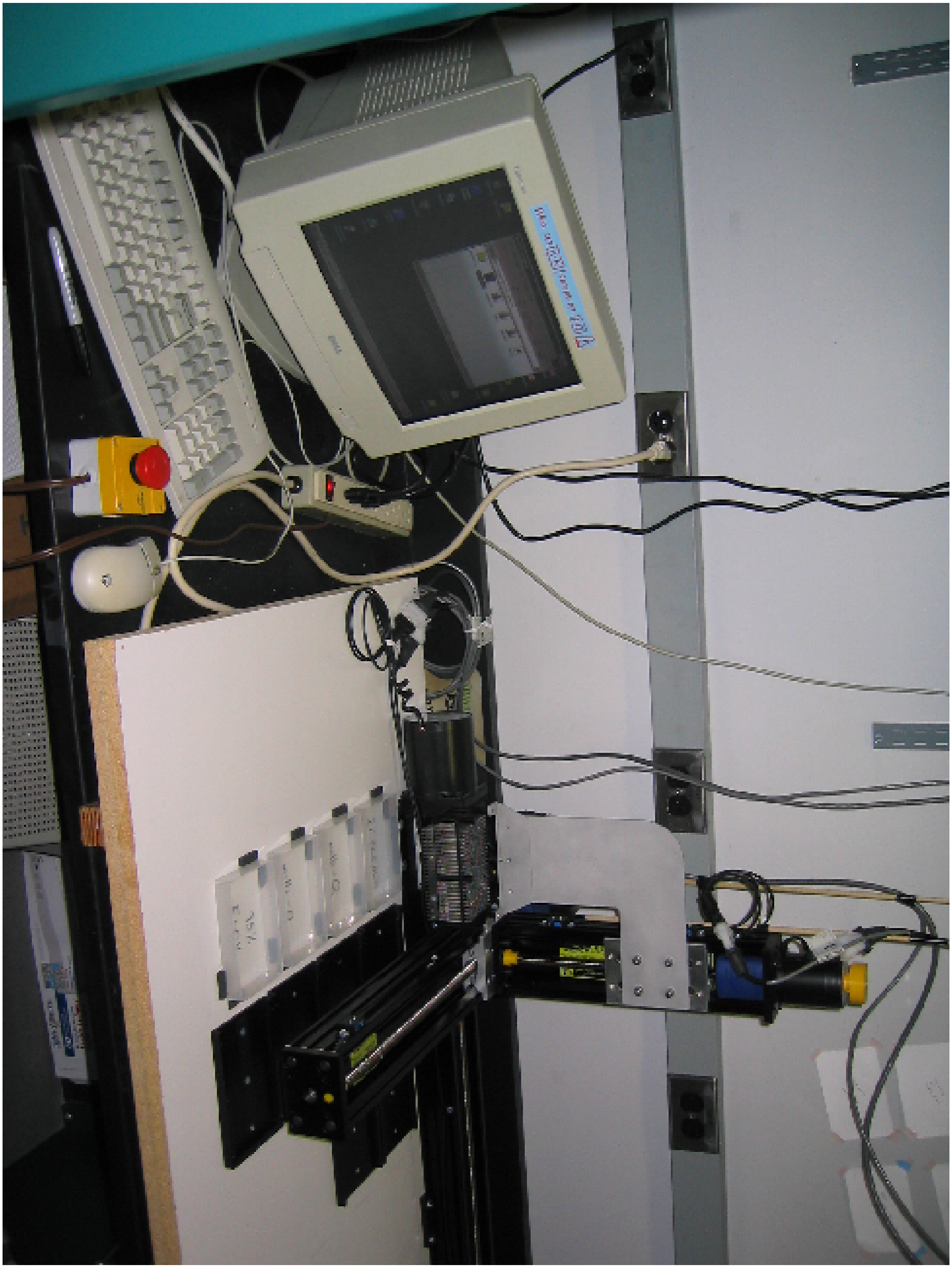

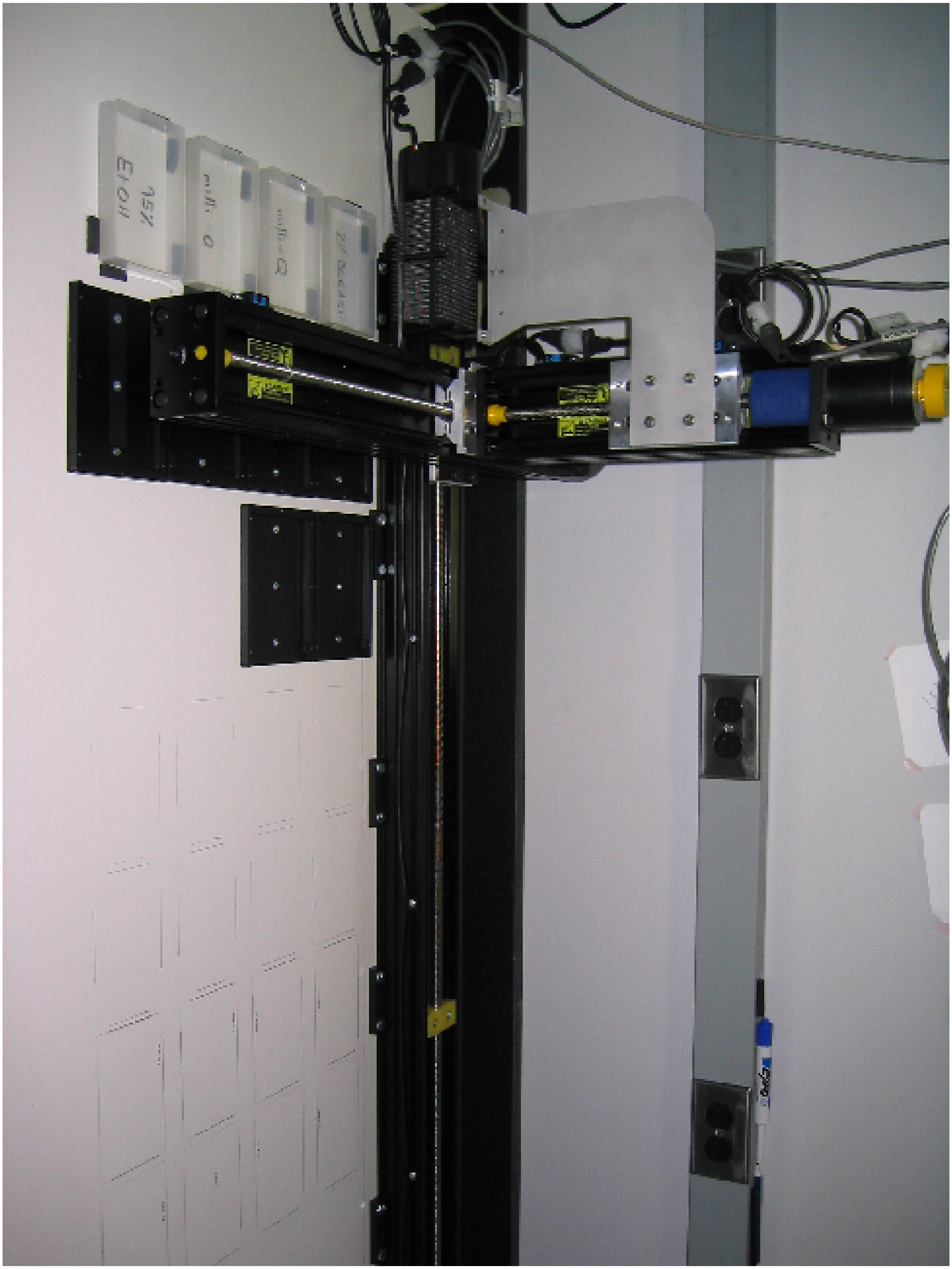

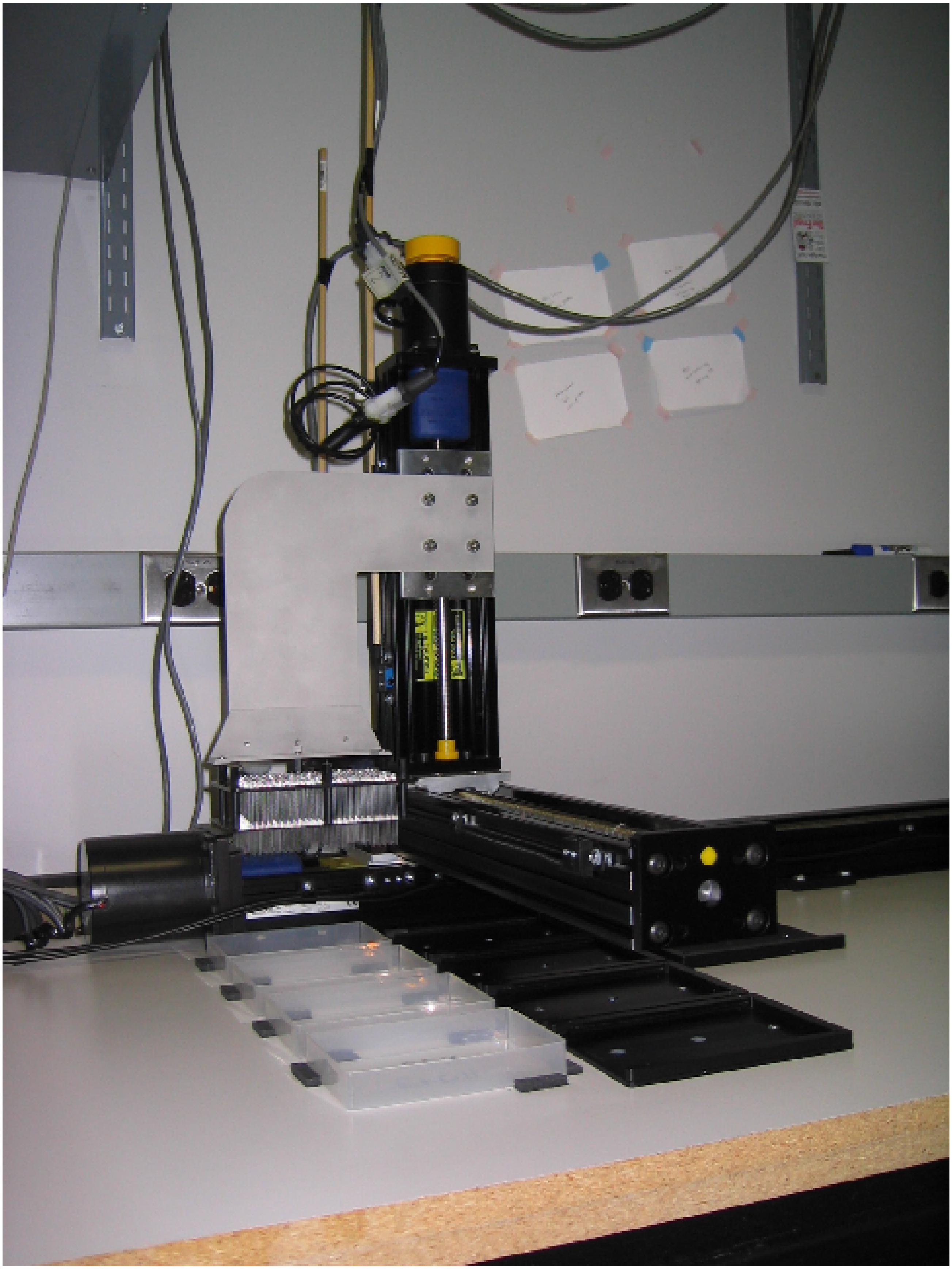

Supplement: Additional data file 6 — This includes a parts list, diagrams, and photographs of the system. [file gb-2005-6-12-r105-S6.pdf]
