# Supplementary figures and images for: A low-cost open-source SNP genotyping platform for association mapping applications
Source: Genome Biol. 2005 Dec 2;6(12):R105. doi: 10.1186/gb-2005-6-12-r105 (PMC1414086; doi:10.1186/gb-2005-6-12-r105)

Probe B  $\log_e(\text{intensity})$

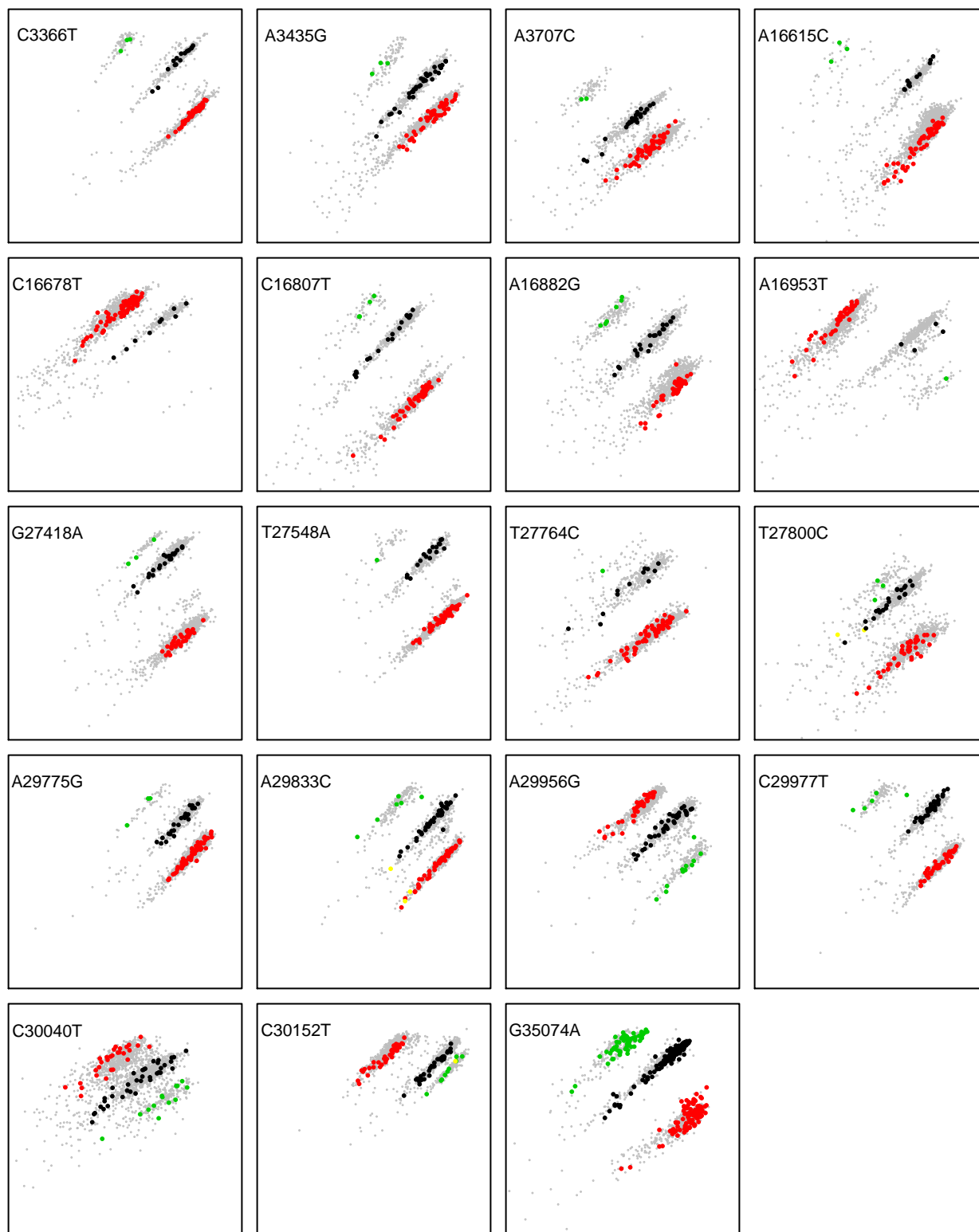

Probe A  $\log_e(\text{intensity})$

Supplement: Additional data file 12 — Each plot displays approximately 2,000 points, representing single D. melanogaster individuals. The points representing individuals assigned genotypes by an OLA assay and by sequencing are colored and large, while the remaining individuals are shown as smaller gray points. Red, major allele homozygote in both OLA and sequencing; black, heterozygote in both OLA and sequencing; green, minor allele homozygote in both OLA and sequencing; yellow, OLA and sequencing yield different genotypes. [file gb-2005-6-12-r105-S12.pdf]
